# Rational Design of Non-Noble Metal Single-Atom Catalysts in Lithium–Sulfur Batteries through First Principles Calculations

**DOI:** 10.3390/nano14080692

**Published:** 2024-04-17

**Authors:** Yang Li, Yao Liu, Jinhui Zhang, Dashuai Wang, Jing Xu

**Affiliations:** 1 Department of Physics, College of Science, Yanbian University, Yanji 133002, China2022010049@ybu.edu.cn (J.Z.); 2 Institute of Zhejiang University-Quzhou, Quzhou 324000, China; 3 Key Laboratory of Biomass Chemical Engineering of Ministry of Education, College of Chemical and Biological Engineering, Zhejiang University, Hangzhou 310027, China

**Keywords:** single-atom catalysts, VS_2_, first-principles calculations, Li–S batteries

## Abstract

Lithium–sulfur (Li–S) batteries with a high theoretical energy density of 2600 Wh·kg^−1^ are hindered by challenges such as low S conductivity, the polysulfide shuttle effect, low S reduction conversion rate, and sluggish Li_2_S oxidation kinetics. Herein, single-atom non-noble metal catalysts (SACs) loaded on two-dimensional (2D) vanadium disulfide (VS_2_) as the potential host materials for the cathode in Li–S batteries were investigated systematically by using first-principles calculations. Based on the comparisons of structural stability, the ability to immobilize sulfur, electrochemical reactivity, and the kinetics of Li_2_S oxidation decomposition between these non-noble metal catalysts and noble metal candidates, Nb@VS_2_ and Ta@VS_2_ were identified as the potential candidates of SACs with the decomposition energy barriers for Li_2_S of 0.395 eV (Nb@VS_2_) and of 0.162 eV (Ta@VS_2_), respectively. This study also identified an exothermic reaction for Nb@VS_2_ and the Gibbs free energy of 0.218 eV for Ta@VS_2_. Furthermore, the adsorption and catalytic mechanisms of the VS_2_-based SACs in the reactions were elucidated, presenting a universal case demonstrating the use of unconventional graphene-based SACs in Li–S batteries. This study presents a universal surface regulation strategy for transition metal dichalcogenides to enhance their performance as host materials in Li–S batteries.

## 1. Introduction

With the growing energy density demand in fields such as electronic devices and electric vehicles, conventional commercial lithium-ion batteries cannot meet the requirements of development. Consequently, scientists are now dedicated to developing new energy storage candidates. Among various battery systems, Li–S batteries are regarded as a promising energy storage candidate based on the abundant reserves, low cost, and non-toxic nature of sulfur, especially their impressively high theoretical specific energy density (2600 W∙h∙kg^−1^) and volumetric energy density (2800 W∙h∙L^−1^) [1,2,3,4]. However, the large-scale commercial application of Li–S batteries is still impeded because of the following reasons: (i) low sulfur utilization resulting from the poor conductivity of the sulfur (S_8_) cathode material and the insoluble discharging products of lower-order lithium polysulfides (LiPSs) such as Li_2_S_2_ and Li_2_S; (ii) the continuous loss of active substances due to the notorious “shuttle effect” of soluble higher-order LiPSs Li_2_S_n_ (4 ≤ *n* ≤ 8) in the charging/discharging process, wherein soluble intermediate higher-order LiPSs species in the electrolyte reach the lithium anode and react with Li metal to form insoluble Li_2_S_n_ (1 ≤ *n* < 4) and deposit on it, which results in the sluggish kinetics of LiPSs, quick reductions in capacity, and cycle stability issues; (iii) the pulverization of the sulfur cathode caused by large volumetric expansion during the lithiation process [5]. In 2D materials, the 100 cycle capacity retention rate using catalytic materials could be increased by about 20% compared to not using catalytic materials; for example, the capacity retention rate of MnO increased from 76.1% to 94.4% after the addition of catalytic materials [6].

In recent years, some strategies have been proposed to address the above problems of Li–S batteries. Particularly, the composite material formed by adding additives to the cathodes of Li–S batteries is expected to solve the problem of low conductivity [7,8,9]. Some framework materials with physical confinement functions are expected to solve the problem of excessive volume changes to the sulfur cathode during charging and discharging processes. Meanwhile, a lot of effort has been invested in developing suitable materials to mitigate the “shuttle effect”. The so-called suitable materials should bind LiPSs to inhibit their migration and accelerate their redox kinetic process, that is, the suitable materials should be materials with anchoring functions [10]. Anchoring materials have been regarded as electrocatalysts which play a crucial role in LiPSs conversion and reduce the reaction barriers, which has attracted great attention. For example, some 2D materials [11,12], including graphene [13,14], MXenes [15], transition metal carbides [16,17], and metal sulfides [18,19], have been elucidated as promising anchoring materials for Li–S batteries due to large specific surface areas and unique physical and chemical properties. Many studies have focused on enhancing the binding of the anchoring material and LiPSs by predicting the adsorption energy based on density functional theory (DFT) simulations. However, most of these 2D materials exhibit incomplete LiPSs conversion to Li_2_S_2_ or Li_2_S due to the sluggish redox reaction kinetics.

Recently, SAC materials consisting of monodispersed transition metal (TM) atoms supported on the surfaces of 2D materials have received much attention. Apart from the maximum metal atom utilization efficiency, SACs can offer outstanding catalytic activity with low metal loading, tunable structure, flexible selectivity, and low cost. In 2021, Cheng’s group proposed that single-atom Ti as the sulfur cathode catalyst could promote battery performance for Li–S batteries, which had the lowest electrochemical barrier to LiPSs reduction/Li_2_S oxidation and the highest catalytic activity. Because of the highly active catalytic center of single-atom Ti on the conductive transport network, high sulfur utilization was achieved with low catalyst loading (1 wt.%) and a high area of sulfur loading (8 mg·cm^−2^) [20,21]. Compared with 2D materials, SACs for Li–S batteries can provide suitable adsorption strength with LiPSs and the exceptional ability to improve reaction kinetics [22,23,24,25]. It can be seen that the adsorption energy between Co and N/G (−7.99 eV) is more negative than those of CoS (−6.39 eV) and CoS_2_ (−7.01 eV) [26]. Among the reported 2D materials in Li–S batteries, 2D transition metal chalcogenides have been widely researched and applied because of their abundant reserves, environmentally friendly nature, and fast reaction kinetics. In particular, T-phase 2D VS_2_ has good intrinsic conductivity compared with FeS_2_, MoS_2_, NiS_2_, NbS_2_, TiS_2_, and ZrS_2_, as well as moderate adsorption strength for Li_2_S_n_ [25,27,28]. For example, to further validate that the adsorption capacities of LiPSs on the surfaces of FeS_2_ were moderate for Li_2_S_n_, the adsorption energies of Li_2_S_4_ on FeS_2_, Ti_3_C_2_, CoP, and graphene surfaces were compared and measured as −4.25, −4.16, −4.91, and −1.21 eV, respectively, indicating a moderate adsorption ability of FeS_2_ [29].

2D VS_2_ as a host material loaded the monodispersed TM atom (hereafter denoted as TM@VS_2_) to constitute SAC materials, which is expected to have the dual effect of suppressing the shuttle effect and accelerating reaction kinetics [22,26,30,31,32]. In this work, 17 unique SACs TM@VS_2_ (TM = 4d and 5d TMs) for sulfur cathodes were constructed and investigated systematically via DFT simulations. By comparing the thermal stability values of 17 TM@VS_2_, the adsorption behavior of S_8_ and five LiPSs intermediates (Li_2_S_n_, *n* = 1, 2, 4, 6, and 8), and the conversion mechanism of Li_2_S_2_ to Li_2_S on the TM@VS_2_, the promising SAC candidates Nb@VS_2_ and Ta@VS_2_ were screened out. This study provided an atomic-level understanding about the reaction mechanism of this composite material TM@VS_2_ in Li–S batteries and clarified how to inhibit the shuttle effect and accelerate the reaction kinetics, which is of significance for the rational design of SACs.

## 2. Methods

All the DFT simulations were performed using the Vienna ab initio simulation package (VASP) [33]. Projector augmented wave (PAW) pseudopotentials and Perdew–Bruke–Ernzerhof (PBE) functions with the generalized gradient approximation (GGA) were employed to investigate the electron–ion interactions and the electron–electron exchange correlations [20,34]. The kinetic energy cutoff was selected as 400 eV for the plane wave basis calculations, the energy convergence was set to 10^−5^ eV/Å, and the force convergence criterion for optimization was set to 0.05 eV/Å. A vacuum space of 20 Å was set to avoid the interactions between the periodic boundaries. The van der Waals interactions were calculated using the DFT-D3 method to provide better accuracy for the adsorption strength of polysulfides with TM@VS_2_. A 4 × 4 × 1 supercell of VS_2_ monolayer was built and a 2 × 2 × 1 K-point was set in the first Brillouin zone for the geometric optimizations and the density of states (DOS) calculation [35]. DOS and electrostatics potential were calculated by DS-PAW software (2023A) integrated in the Device Studio program [36]. In addition, ab initio molecular dynamics (AIMD) simulations of Pd@VS_2_, Rh@VS_2_, and Ta@VS_2_ were carried out to confirm material stabilities on the 8 × 4 × 1 supercell at 298.15 K within 10 ps. The decomposition properties of Li_2_S were determined using the climbing-image nudged elastic band (CI-NEB) method. The SACs among 17 single TM atoms loaded on the VS_2_ monolayer were first screened by calculating the adsorption energy following Equation (1):

(1) $$E_{{ad}_{1}}=E_{{TM@VS}_{2}}-E_{TM}-E_{{VS}_{2}}$$

where $E_{{TM@VS}_{2}}$ and $E_{{VS}_{2}}$ are the energies of loaded single atoms and the bare VS_2_ monolayer, respectively. E_TM_ is the energy per single atom in the metallic phase. A more negative value of $E_{{ad}_{1}}$ represents a stronger adsorption.

The adsorption energies $E_{{ad}_{2}}$ of Li_2_S_n_ and S_8_ molecules adsorbed on the VS_2_ and TM@VS_2_ monolayer are calculated based on the following Equation (2):

(2) $$E_{{ad}_{2}}=E_{total}-E_{sub}-E_{mol}$$

where $E_{total}$ represents the total energy of adsorbed system Li_2_S_n_ (or S_8_) on TM@VS_2_ or VS_2_ monolayer, $E_{sub}$ represents the energy of TM@VS_2_ or VS_2_ monolayer, and $E_{mol}$ represents the energy of Li_2_S_n_ (*n* = 1, 2, 4, 6, 8) and the S_8_ molecule.

To visualize the charge transfer of Li_2_S_n_ molecules on TM@VS_2_ monolayers, the charge density differences (CDDs) of the adsorbed configurations were displayed by calculating from the following Equation (3):

(3) $$∆\rho =\rho_{total}-\rho_{sub}-\rho_{mol}$$

where $\rho_{total}$, $\rho_{TM}$, and $\rho_{mol}$ represent the charge density of the whole adsorbed system, TM@VS_2_ or VS_2_ monolayer, and Li_2_S_n_ and S_8_ molecule, respectively.

The Gibbs free energies can be defined as follows (4):

(4) ∆G=∆E+∆ZPE−T∆S

where ∆E represents the electronic energy of the product minus that of the reactant, and ∆ZPE and ∆S represent the zero-point vibrational energy and entropy change at the standard temperature and pressure, respectively.

## 3. Results and Discussion

### 3.1. Structure and Stability of the TM@VS_2_ SACs

**Figure 1:**
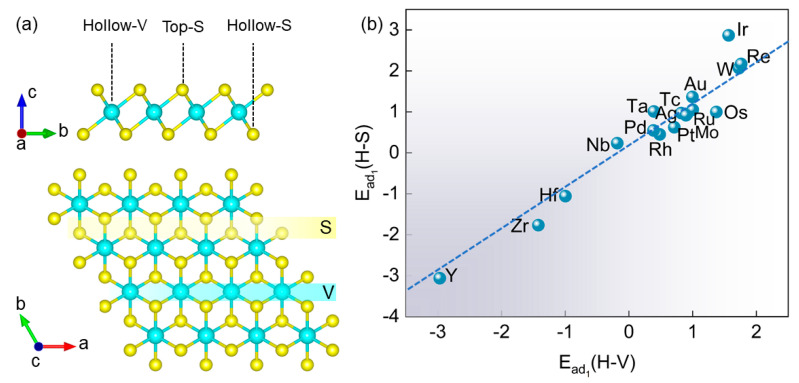
(**a**) Top and side views of VS_2_ monolayer with three different adsorption sites for single atoms. (**b**) The adsorption energies of single atoms at H-V and H-S sites. The dashed line indicates the equal adsorption energies of H-V and H-S sites.

The configuration of T-phase VS_2_ is shown in Figure 1a. There are three possible sites where a single TM atom can deposit on the surface of the VS_2_ monolayer, including Hollow-V (H-V), Top-S (T-S), and Hollow-S (H-S). In order to screen the most suitable loaded single TM atoms on the VS_2_ monolayer to constitute SACs, the adsorption energies of 17 4d and 5d TM atoms loaded on the VS_2_ surface at three possible sites were calculated based on Equation (1). Remarkably, the adsorption of single atoms at the T-S site after the optimization became higher than the H-S or H-V sites, such that the adsorption energies were higher than those of the H-S or H-V sites, even to positive values (about 0.5–7.1 eV), which indicated that the adsorption of the T-S site was unstable thermodynamically. Therefore, all the TM@VS_2_ at the T-S site will not be considered in the following. The adsorption energies at H-V and H-S sites are depicted in Figure 1b. The results revealed that the single TM atoms of four species (Y, Zr, Nb, and Hf) were strong in affinity and stably adsorbed on the VS_2_ surface due to the negative adsorption energies. In addition, Pd@VS_2_, Rh@VS_2_, and Ta@VS_2_ displayed slightly positive adsorption energies, suggesting their comparatively weaker interactions. Further, the thermal stability of Pd@VS_2_, Rh@VS_2_, and Ta@VS_2_ was considered by AIMD simulation. The final configurations of Pd@VS_2_, Rh@VS_2_, and Ta@VS_2_ after AIMD simulations are depicted in Figure S1a–c. The simulation profiles revealed a consistent trend of converging energy, with the structures maintaining their original coordination environment even after 10 ps. No structural deformations or atomic clustering were observed, demonstrating the thermal stability of Pd@VS_2_, Rh@VS_2_, and Ta@VS_2_. Furthermore, Figure 1 shows that Hf, Rh, Y, and Zr atoms were preferentially adsorbed at the H-S site, while Pd, Ta, and Nb atoms preferred the H-V site. For a single TM atom, adsorption at the H-V and H-S sites on the VS_2_ surface site are comparable, and the more favorable sites can be screened by calculating the COHP of TM–S bonds at the H-S and H-V sites based on a comparison of adsorption energies, with sites showing more negative adsorption energies being considered optimal. Compared to the other site, Nb–V, Ta–V, Zr–S, and Rh–S demonstrate higher ICOHP values, indicating stronger bonding strength with single atoms (Figure S2). This disparity in site behavior arises from differences in the types of single atoms and the adsorption strengths of various adsorption sites on the VS_2_ substrate. The lowest adsorption energy corresponds to the most stable configuration, as shown in Figure S3a. Furthermore, different coordination numbers have different geometric structures and chemical properties. The optimized configurations of the SACs substantiate a TM–S with three coordinates on the VS_2_ substrate. Typically, compared to four coordinates in TM–N_4_–graphene [37,38], TM–S with three coordinates on a VS_2_ surface has fewer coordination numbers, resulting in a single atom with more unbonded electrons, and these sites can adsorb and activate reactants, facilitating the reaction.

**Figure 2:**
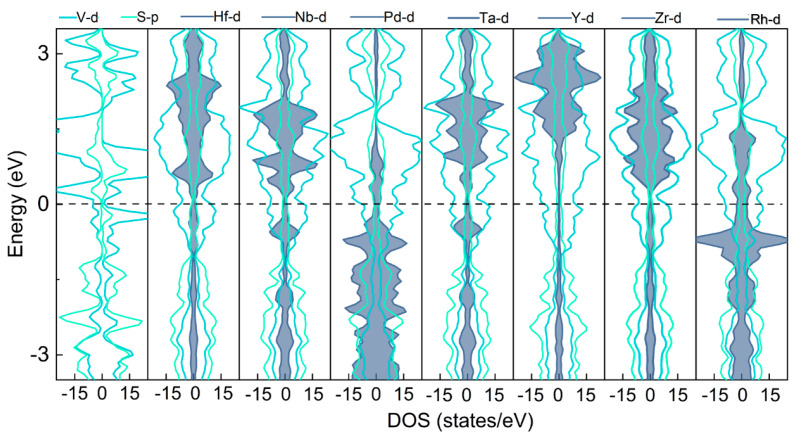
PDOS of VS_2_ and TM@VS_2_ (TM = Hf, Nb, Pd, Ta, Y, Zr, or Rh, where the d orbitals of each TM atom are multiplied by 10).

In addition, in order to better understand the bonding properties between the TM atom and VS_2_ monolayer, the projected density of states (PDOS) of TM@VS_2_ was calculated based on fully relaxed configurations, as shown in Figure 2. The results provided compelling evidence of significant d-p orbital hybridization between the TM and S atoms, indicating that there were stable covalent bonds between TM and S atoms. Furthermore, a continuous state density across the Fermi level indicated that these TM@VS_2_ were metallic. This observation carried crucial implications for enhancing the conductivity of the cathode. Specifically, the adsorption energy of the TM and VS_2_ monolayer is often linked to the electron state’s density, as the d orbitals of Y, Zr, and Hf exhibit more electron states above the Fermi level than other metal atoms, therefore exhibiting stronger adsorption energy.

### 3.2. Anchoring Effect of TM@VS_2_ SACs

**Figure 3:**
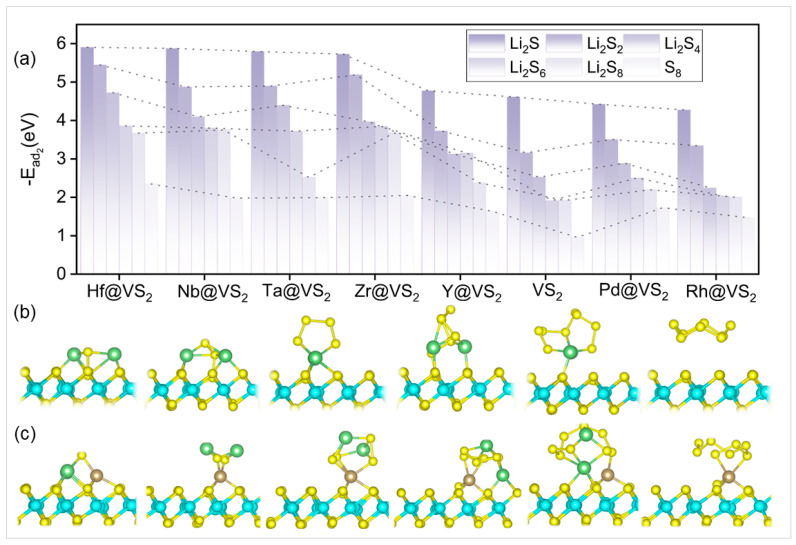
(**a**) The difference in adsorption energies between Li_2_S_n_/S_8_ on VS_2_ and TM@VS_2_. The adsorption configurations of Li_2_S_n_/S_8_ on (**b**) VS_2_ and (**c**) Ta@VS_2_.

The primary objective of catalysts of the sulfur cathode for Li–S batteries is to enhance the immobilization of sulfur and polysulfides. To investigate the anchoring effect of TM@VS_2_, the adsorption energies of VS_2_ and the TM@VS_2_ monolayer for the intermediate polysulfides Li_2_S_n_ (*n* = 1, 2, 4, 6, 8) and S_8_ during charge–discharge processes were calculated based on Equation (2). The values of all adsorption energies were shown in Figure 3a and Table S1. The adsorption energies of Li_2_S_n_ and S_8_ on the surface of VS_2_ were as follows: Li_2_S (−4.613 eV), Li_2_S_2_ (−3.158 eV), Li_2_S_4_ (−2.525 eV), Li_2_S_6_ (−1.915 eV), Li_2_S_8_ (−1.918 eV), and S_8_ (−0.957 eV). The corresponding optimal configurations of VS_2_ are given in Figure 3b. Nb shows obvious higher electrostatic potential than that of Pd (Figure S4), which is an electrophilic center to adsorb polysulfides favorably in the reaction process, and the adsorption strength was enhanced to inhibit the shuttle effect effectively compared to the VS_2_ substrate. The corresponding optimal configurations of Ta@VS_2_, Pd@VS_2_, Hf@VS_2_, Rh@VS_2_, Y@VS_2_, Nb@VS_2_, and Zr@VS_2_ are given in Figure 3c and Figure S3b–g. There is no significant structural deformation observed after the introduction of single atoms. The results indicated that most of the adsorption energies were enhanced after the introduction of TM single atoms on the VS_2_ monolayer, where the adsorption energies of Ta@VS_2_, Hf@VS_2_, Y@VS_2_, Nb@VS_2_, and Zr@VS_2_ could all be enhanced for Li_2_S_2_, Li_2_S_6_, Li_2_S_8_, and S_8_ compared with VS_2_, which exhibited the sulfur immobilization ability of these non-noble metal SACs. However, noble metal atoms Pd and Rh enhanced adsorption ability partially for S_8_ and LiPSs compared with VS_2_. In addition, the adsorption strengths of these five non-noble metal SACs for soluble higher-order LiPSs Li_2_S_n_ (4 ≤ *n* ≤ 8) were significantly higher than the interactions between common electrolyte components and soluble intermediates [39]; for example, the adsorption energy of 1,3-dioxolane (DOL) for Li_2_S_6_ was −0.75 eV, and that of 1,2-dimethoxyethane (DME) was −0.77 eV. Differently, the adsorption energies for Li_2_S_6_ were −3.802 eV (Nb@VS_2_) and −3.860 eV (Hf@VS_2_), which effectively prevented the shuttle effect of LiPSs. Therefore, non-noble metal atoms Hf, Nb, Ta, Y, and Zr loaded on VS_2_ monolayer have the potential to enhance sulfur immobilization capability compared to noble metal atoms Pd and Rh.

Taking Ta@VS_2_ as an example, the optimized adsorption configurations are shown in Figure 3b, which reveals that Ta@VS_2_ forms both Li–S and Ta–S bonds during the adsorption process. Compared to the Li–S bond on the surface of VS_2_, the presence of more bonding sites and species offers the potential to enhance the adsorption effect for TM@VS_2_. To gain a further insight into the mechanisms between the adsorption strength and bonding, the CDD between TM@VS_2_ and the adsorbed intermediates were investigated, and the results are shown in Figure S5 for VS_2_, Pd@VS_2_, Hf@VS_2_, Rh@VS_2_, Y@VS_2_, Ta@VS_2_, Nb@VS_2_, and Zr@VS_2_, respectively. The results revealed that the electron transfer primarily occurred between Li atoms and S atoms on the surface of VS_2_ monolayer. However, for non-noble SACs such as Hf@VS_2_, Nb@VS_2_, Ta@VS_2_, Zr@VS_2_, and Y@VS_2_, the electron transfer occurred not only between Li atoms and S atoms but also between the single TM atoms and the S atoms in Li_2_S_n_, forming TM–S bonds. This implies there was a more significant electron transfer between TM@VS_2_ and the S atoms in Li_2_S_n_ clusters. For all the non-noble SACs, the electron transfer from S_8_ to Li_2_S gradually increases, and the largest charge transfer occurred between the SACs and Li_2_S, corresponding to the strongest adsorption energy. The enhanced adsorption of S_8_ can also be attributed to the redistribution of charge between individual TM atoms. Furthermore, there exists a competitive bonding interaction between Li–TM@VS_2_ and Li–S/S–S, wherein TM atoms interact with S atoms to form charge density accumulations during chemical bonding. Meanwhile, varying degrees of charge loss are observed in the S–S/Li–S bonds within the Li_2_S_n_ molecules. As lithiation progresses, the strength of Li–S bonds within the Li_2_S_n_ clusters weakened, while the adsorption capability of Li_2_S_n_ on TM@VS_2_ increased. In addition, the weakened Li–S bonds are expected to promote the reaction kinetics of Li_2_S_n_ conversion.

### 3.3. The Catalytic Mechanism of TM@VS_2_

**Figure 4:**
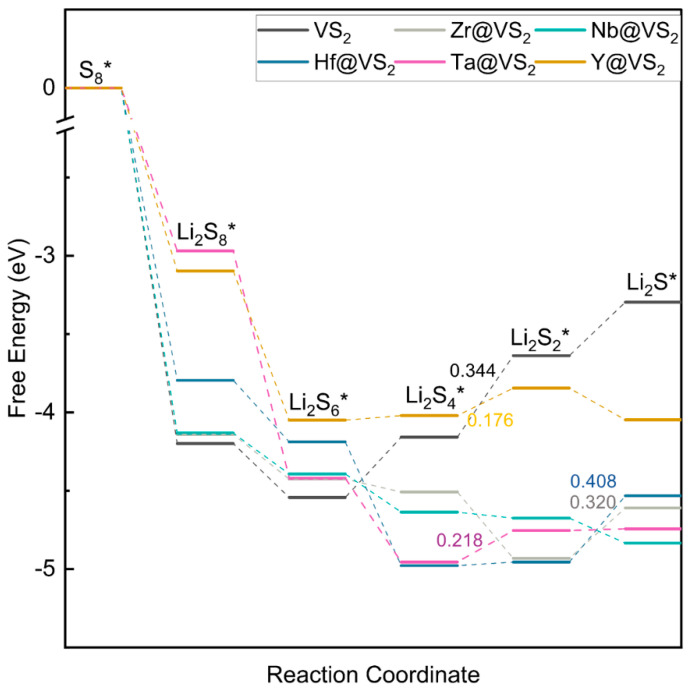
Gibbs free energy of S_8_-to-Li_2_S reaction on VS_2_ and TM@VS_2_ (TM = Y, Zr, Hf, Nb, Ta. The * was stand for absorbed).

To ascertain the catalytic performance of TM@VS_2_ (TM = Y, Zr, Hf, Nb, Ta, Rh, Pd) in the sulfur reduction reaction, the Gibbs free energy for the conversion of S_8_ to Li_2_S was calculated. The energy landscape was depicted in Figure 4 and Figure S6. The research results revealed that the Gibbs free energies of the reaction-determining step were 0.344 (VS_2_), 0.176 (Y@VS_2_), 0.320 (Zr@VS_2_), 0.408 (Hf@VS_2_), 0.218 (Ta@VS_2_), and 0.154 eV (Pd@VS_2_), respectively. The Gibbs free energy of Rh@VS_2_ and Pd@VS_2_ are shown in Figure S6. The reactions of Rh@VS_2_ and Nb@VS_2_ were exothermic, indicating that the reactions proceeded readily. According to the above, Hf@VS_2_ has a higher Gibbs free energy, which is unfavorable for the reaction thermodynamics. Therefore, Y@VS_2_, Pd@VS_2_, Zr@VS_2_, and Ta@VS_2_ accelerated the sulfur lithiation reaction compared to VS_2_, and Nb@VS_2_ and Rh@VS_2_ experienced exothermic reactions, which highlighted their relatively strong catalytic activity in the sulfur reduction reaction.

**Figure 5:**
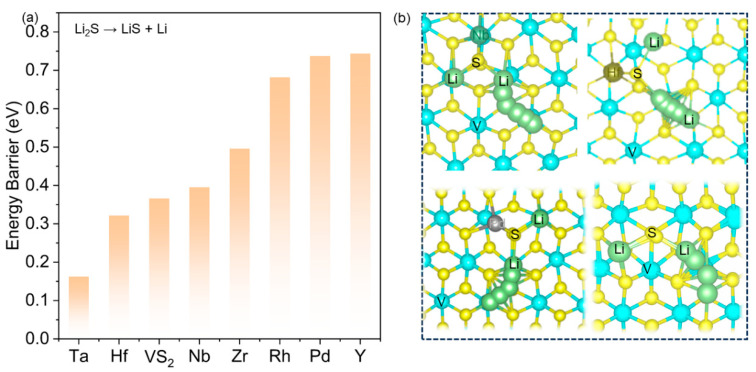
(**a**) Decomposition energy barriers of Li_2_S on VS_2_ and TM@VS_2_. Li_2_S decomposition pathway on (**b**) Nb@VS_2_, Hf@VS_2_, Pd@VS_2_, and VS_2_.

**Figure 6:**
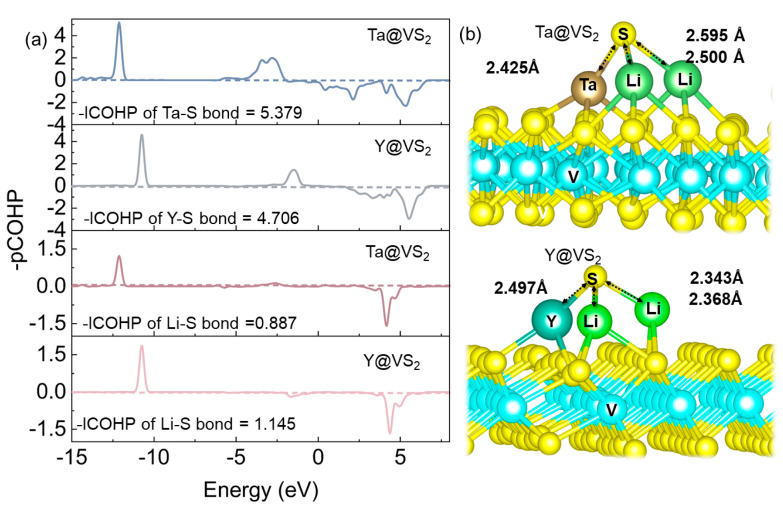
(**a**) COHP diagram of the TM–S and Li–S bonds in Li_2_S on Ta@VS_2_ and Y@VS_2_. (**b**) The Li_2_S adsorption configurations on Ta@VS_2_ and Y@VS_2_.

In Li–S batteries, the generation of Li_2_S as the final discharge product causes the challenge of sluggish reaction kinetics due to its low electronic conductivity and Li^+^ diffusion rates [40]. A crucial approach to address the slow oxidation rate during Li–S battery charging is to decrease the decomposition energy barrier of Li_2_S. Therefore, it is necessary to evaluate the catalytic oxidation performance of TM@VS_2_ for the charging process by examining the decomposition energy barriers of Li_2_S. The calculated results are shown in Figure 5 and Figure S7, and significant differences were observed in Li_2_S decomposition energy barriers on various surfaces. The decomposition energy barrier of Li_2_S on the VS_2_ surface was 0.366 eV. The corresponding decomposition energy barriers of Li_2_S on TM@VS_2_ were 0.162 (Ta@VS_2_), 0.322 (Hf@VS_2_), 0.396 (Nb@VS_2_), 0.496 (Zr@VS_2_), 0.682 (Rh@VS_2_), 0.736 (Pd@VS_2_), and 0.744 eV (Y@VS_2_). The results indicated that TM@VS_2_ (TM = Nb, Ta, Hf) could significantly reduce the decomposition energy barrier of Li_2_S compared to the VS_2_ surface. Among all the investigated seven TM@VS_2_, Ta@VS_2_ displayed the lowest decomposition energy barrier. This could be attributed to the formation of chemical bonds between the Ta and the S of Li_2_S, weakening the Li–S bonds and, therefore, lowering the decomposition energy barrier of Li_2_S. Taking this into account, crystal orbital Hamilton population (COHP) analysis was introduced to explore the bonding strength of Li–S and TM–S bonds in Li_2_S on TM@VS_2_, as shown in Figure 6. The COHPs of Ta@VS_2_ and Pd@VS_2_ were selected for comparative analysis as an example. Compared to Pd@VS_2_, the bonding state of the Ta–S bond below the Fermi level is significantly lower in Ta@VS_2_–Li_2_S than that of Y–S, with ICOHP values of 5.379 and 4.706 eV, respectively. This is accompanied by an increase in bond length from 2.425 Å for Ta–S to 2.497 Å for Y–S. Meanwhile, the Li–S bonds in the adsorbed state of Li_2_S exhibit an opposite ICOHP trend, with the Li–S bond in Ta@VS_2_–LiS at 0.887 and in Y@VS_2_–Li_2_S at 1.145. The corresponding Li–S bond lengths are 2.595 and 2.500 Å for Ta@VS_2_–Li_2_S and 2.343 and 2.368 Å for Y@VS_2_–Li_2_S. This indicates a significant weakening of the Li–S bonds in Li_2_S on Ta@VS_2_, promoting the catalytic oxidation of Li_2_S and aligning with the trend in decomposition energy barriers. Benefiting from high electronic conductivity and excellent bifunctional catalytic ability, Nb@VS_2_ and Ta@VS_2_ can effectively accelerate the Li_2_S_n_ conversion reaction and improve the electrode kinetics of Li–S batteries.

## 4. Conclusions

In summary, this study presents a systematic first-principles calculation of seven VS_2_-based SACs as host materials of the cathode for Li–S batteries. The calculation results show that among the seven selected monatomic catalysts, two noble metals Pd and Rh are removed, and five non-noble metals catalysts are obtained. By comparing the adsorption properties, metal properties, and reaction kinetics, it is found that the addition of Nb and Ta into VS_2_ has the potential to improve the performance of Li–S batteries. This enhancement is mainly attributed to the metallic characteristics of TM@VS_2_, which improve the electronic conductivity of the sulfur cathode. The enhanced adsorption of intermediate discharge products helps suppress the dissolution and shuttle effect of lithium polysulfides. Additionally, the lower reaction-determining step and reduced energy barrier for Li_2_S decomposition contribute to accelerated reaction kinetics. This work provides a universal approach for the atom-scale modification of transition metal sulfides, offering valuable insights and guidance for the development of sustainable and efficient Li–S battery technologies.

## Data Availability

Data are contained within the article and Supplementary Materials.

## Supplementary Materials

The following supporting information can be downloaded at https://www.mdpi.com/article/10.3390/nano14080692/s1. Figure S1. The final configurations of (a) Pd@VS_2_, (b) Rh@VS_2_ and (c) Ta@VS_2_. Figure S2. The ICOHP of TM-S bonds (TM@VS_2_, TM = Nb, Ta, Zr, Rh) for two kind of different site (H-V, H-S). Figure S3. (a) The optimal configurations of TM@VS_2_ (TM = Pd, Hf, Rh, Y, Nb, Zr). (b–g) Optimal configurations of Li_2_S_n_ and S_8_ on TM@VS_2_ (TM = Pd, Hf, Rh, Y, Nb, Zr). Figure S4. The electrostatics potential of (a) VS_2_, (b) Nb@VS_2_, (c) Pd@VS_2_ by DS-PAW method, respectively. Figure S5. Differential charge density of Li_2_S, Li_2_S_6_ and S_8_ on (a) VS_2_, (b) Pd@VS_2_, (c) Hf@VS_2_, (d) Rh@VS_2_, (e) Y@VS_2_, (f) Ta@VS_2_, (g) Nb@VS_2_ and (h) Zr@VS_2_, the iso-surface is set to 0.0025 e/A^3^ (Orange region indicates electron concentration and green region indicates electron deficiency). Figure S6. Gibbs Free Energy of S_8_–to–Li_2_S reaction on TM@VS_2_ (TM = Rh, Pd). Figure S7. Decomposition energy Barriers of Li_2_S on TM@VS_2_. Table S1. The adsorption energies of polysulfides on TM@VS_2_ (The units is electron volts (eV)). Table S2. The optimized coordinates of polysulfides on TM@VS_2_.
