# Supplementary material for: Rational Design of Non-Noble Metal Single-Atom Catalysts in Lithium–Sulfur Batteries through First Principles Calculations
Source: Nanomaterials (Basel). 2024 Apr 17;14(8):692. doi: 10.3390/nano14080692 (PMC11053660; doi:10.3390/nano14080692)
Supplement: Supplementary file 1 [file nanomaterials-14-00692-s001.zip › nanomaterials-2946898-supplementary.pdf]

Supplementary Materials

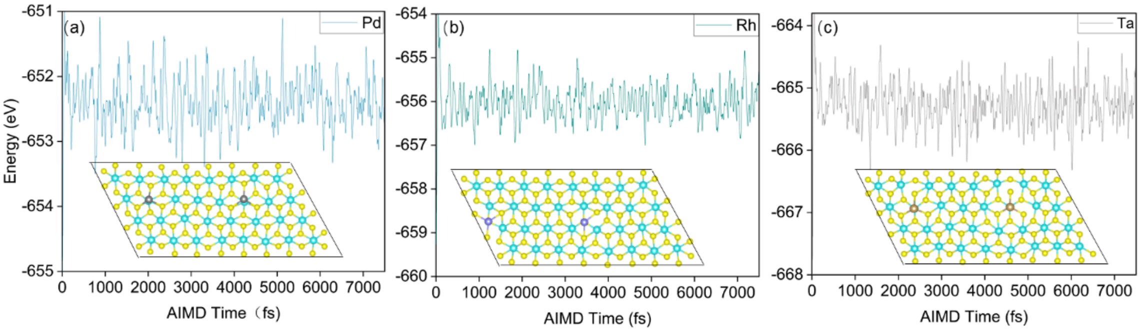

Figure S1 The final configurations of (a) Pd@VS_2, (b) Rh@VS_2 and (c) Ta@VS_2

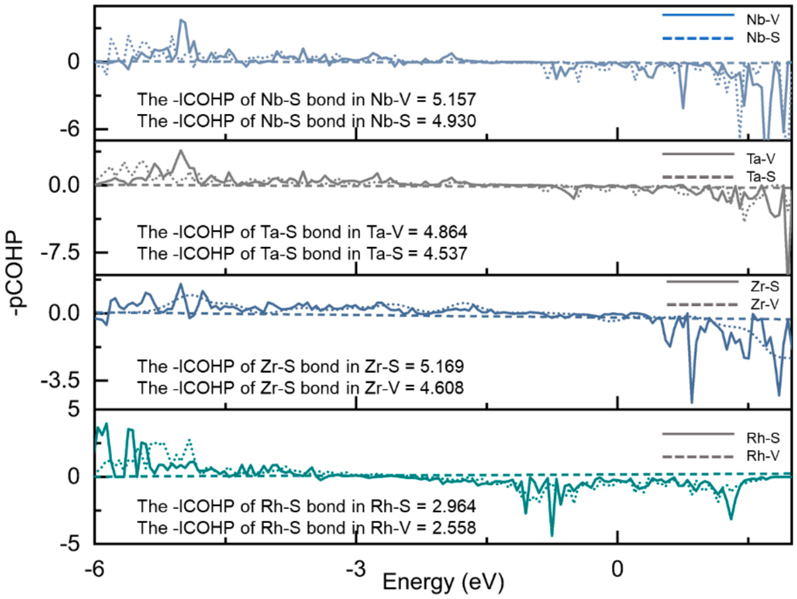

Figure S2 The ICOHP of TM-S bonds (TM@VS_2, TM=Nb, Ta, Zr, Rh) for two kind of different site (H-V, H-S).

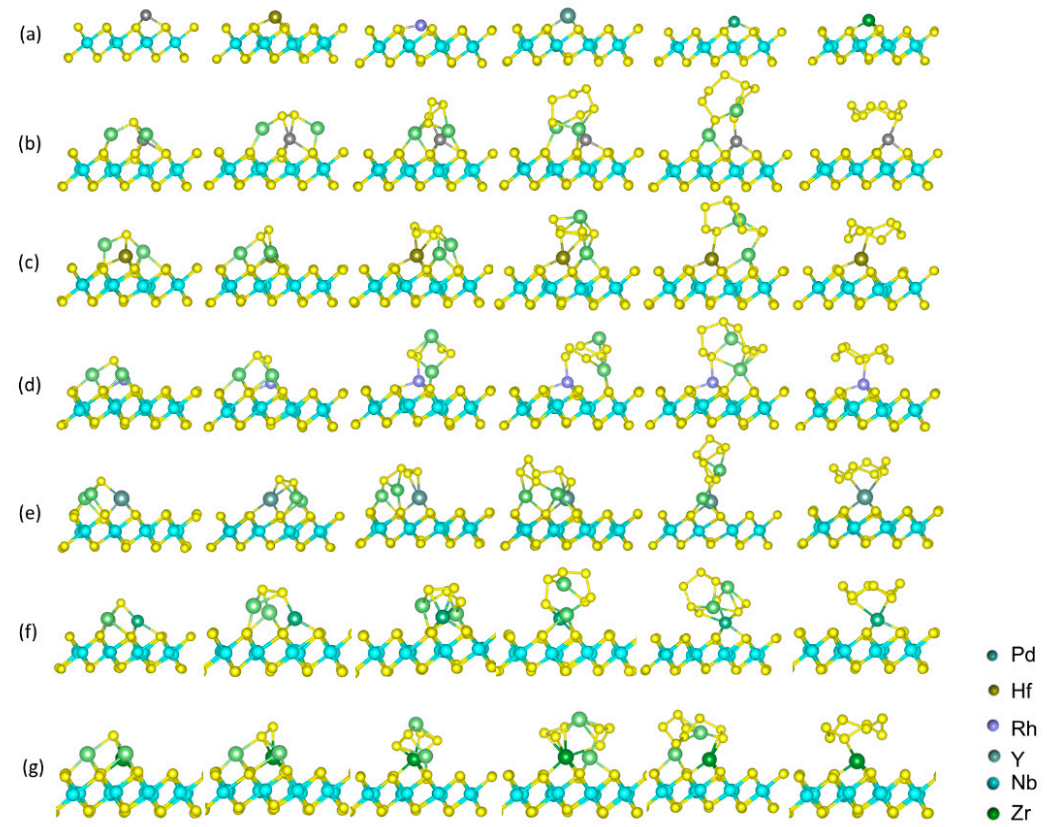

Figure S3 (a) The optimal configurations of TM@VS_2 (TM=Pd, Hf, Rh, Y, Nb, Zr). (b-g) Optimal configurations of Li_2S_n and S_8 on TM@VS_2 (TM=Pd, Hf, Rh, Y, Nb, Zr).

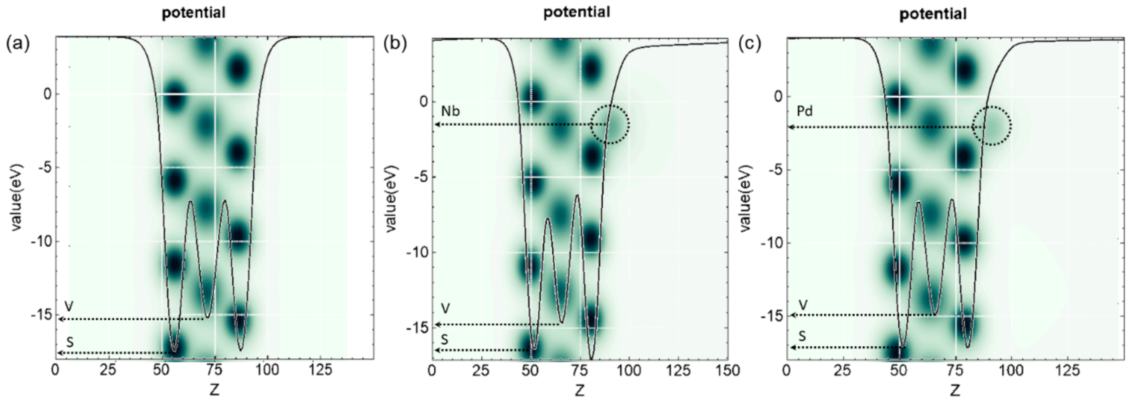

Figure S4 The electrostatics potential of (a) VS_2, (b) Nb@VS_2, (c) Pd@VS_2 by DS-PAW method, respectively.

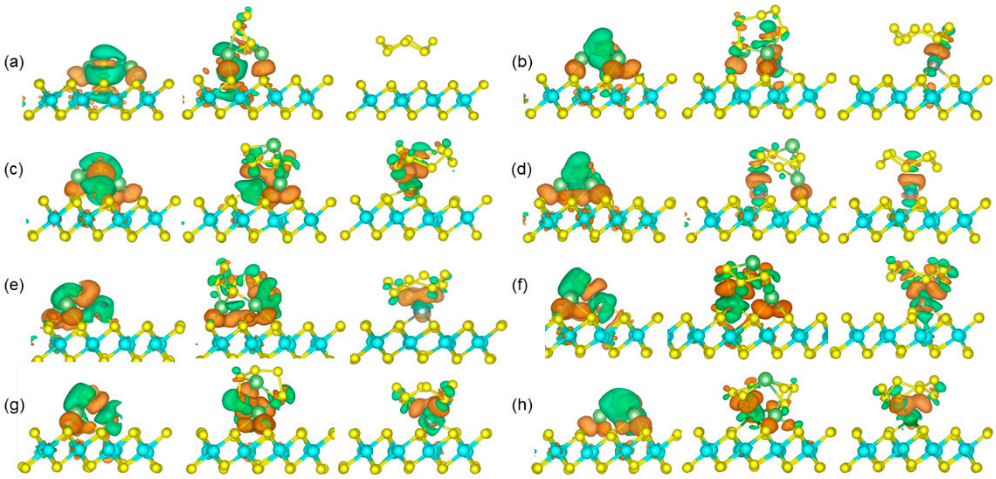

Figure S5 Differential charge density of Li_2S, Li_2S_6 and S_8 on (a) VS_2, (b) Pd@VS_2, (c) Hf@VS_2, (d) Rh@VS_2, (e) Y@VS_2, (f) Ta@VS_2, (g) Nb@VS_2 and (h) Zr@VS_2, the iso-surface is set to 0.0025 e/A^3 (Orange region indicates electron concentration and green region indicates electron deficiency).

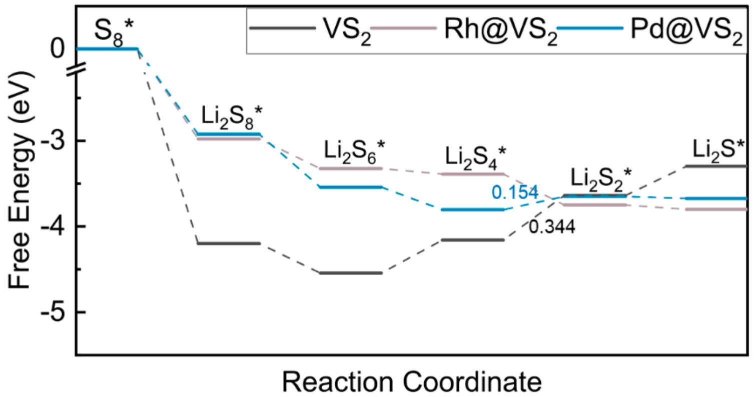

Figure S6 Gibbs Free Energy of S_8–to–Li_2S reaction on TM@VS_2 (TM = Rh, Pd).

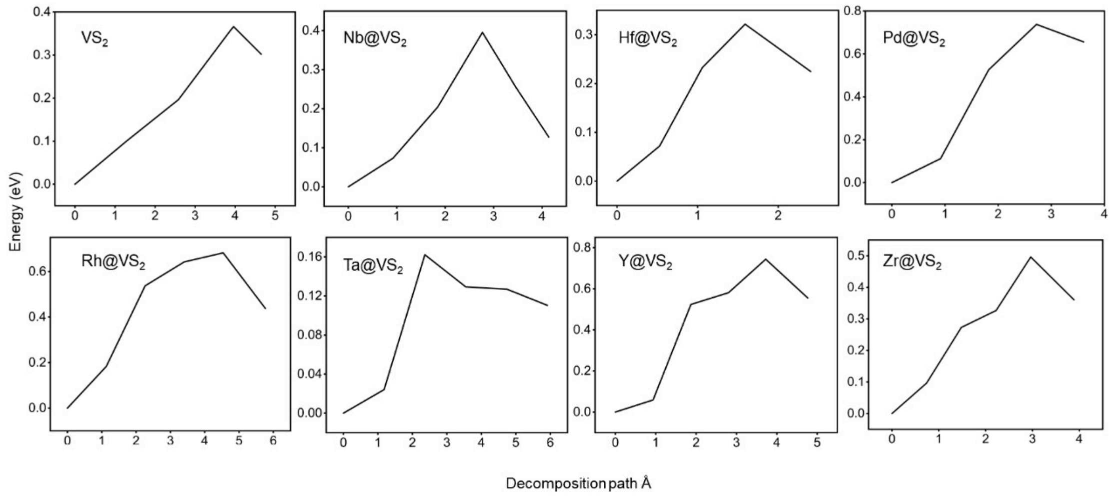

Figure S7 Decomposition energy Barriers of Li_2S on TM@VS_2.

Table S1 The adsorption energies of polysulfides on TM@VS_2 (The units is electron volts (eV))

	Nb@VS_2	Hf@VS_2	Pd@VS_2	Rh@VS_2	Zr@VS_2	Ta@VS_2	Y@VS_2
Li_2S	-5.876	-6.002	-4.423	-4.276	-5.728	-5.795	-4.774
Li_2S_2	-4.873	-5.446	-3.506	-3.344	-5.190	-4.895	-3.727
Li_2S_4	-4.098	-4.722	-2.888	-2.246	-3.966	-4.395	-3.130
Li_2S_6	-3.802	-3.859	-2.506	-2.037	-3.845	-3.721	-3.152
Li_2S_8	-3.752	-3.668	-2.197	-2.005	-3.675	-2.519	-2.376
S_8	-1.981	-2.348	-1.730	-1.469	-2.049	-1.989	-1.637

Table S2 The optimized coordinates of polysulfides on TM@VS_2.

Nb@VS_2			
	1		
	12.884	0	0
	-6.442	11.15787	0
	0	0	17.8775
	V	S	Nb
	16	32	1
Direct			
	0.202506	0.200733	0.362485
	0.478078	0.224128	0.357515
	0.712947	0.221658	0.358734
	0.950177	0.224342	0.357518
	0.189189	0.434507	0.359072
	0.449384	0.434496	0.359041
	0.71287	0.461723	0.358722
	0.953109	0.462012	0.35869

	0.189043	0.72498	0.359098
	0.459918	0.715284	0.361114
	0.739869	0.724774	0.359211
	0.950252	0.69658	0.357691
	0.203049	0.972795	0.362605
	0.44942	0.985254	0.359185
	0.739929	0.98561	0.359146
	0.974149	0.972076	0.362673
	0.042322	0.132483	0.441543
	0.126284	0.048317	0.274444
	0.294453	0.132633	0.441722
	0.374353	0.05419	0.275663
	0.545514	0.137997	0.443568
	0.623975	0.043857	0.286469
	0.79682	0.138166	0.443645
	0.884003	0.054225	0.27582
	0.036616	0.378066	0.443541
	0.120271	0.290436	0.275678
	0.289406	0.374691	0.441485
	0.374323	0.290475	0.27564
	0.545523	0.37795	0.443506
	0.63057	0.300572	0.273522
	0.792913	0.381656	0.442948
	0.874148	0.300663	0.273478
	0.036583	0.629192	0.443695
	0.130936	0.550758	0.286529
	0.273354	0.621942	0.449263
	0.374544	0.544987	0.289629
	0.552669	0.621816	0.449228
	0.623934	0.550687	0.286523
	0.796708	0.629086	0.443729
	0.874243	0.544305	0.273545
	0.042489	0.880442	0.441689
	0.120311	0.800393	0.275844
	0.289416	0.885184	0.441569
	0.374532	0.800011	0.289626
	0.552734	0.901509	0.449014
	0.629753	0.800148	0.289591
	0.799897	0.885226	0.4415
	0.884039	0.800272	0.27583
	0.459686	0.715183	0.498776

Hf@VS_2			
	1		
	12.884	0	0
	-6.442	11.15787	0
	0	0	17.8775
	V	S	Hf
	16	32	1
Direct			
	0.202506	0.200733	0.362485
	0.478078	0.224128	0.357515
	0.712947	0.221658	0.358734
	0.950177	0.224342	0.357518
	0.189189	0.434507	0.359072
	0.449384	0.434496	0.359041
	0.71287	0.461723	0.358722
	0.953109	0.462012	0.35869
	0.189043	0.72498	0.359098
	0.459918	0.715284	0.361114
	0.739869	0.724774	0.359211
	0.950252	0.69658	0.357691
	0.203049	0.972795	0.362605
	0.44942	0.985254	0.359185
	0.739929	0.98561	0.359146
	0.974149	0.972076	0.362673
	0.042322	0.132483	0.441543
	0.126284	0.048317	0.274444
	0.294453	0.132633	0.441722
	0.374353	0.05419	0.275663
	0.545514	0.137997	0.443568
	0.623975	0.043857	0.286469
	0.79682	0.138166	0.443645
	0.884003	0.054225	0.27582
	0.036616	0.378066	0.443541
	0.120271	0.290436	0.275678
	0.289406	0.374691	0.441485
	0.374323	0.290475	0.27564
	0.545523	0.37795	0.443506
	0.63057	0.300572	0.273522
	0.792913	0.381656	0.442948
	0.874148	0.300663	0.273478
	0.036583	0.629192	0.443695
	0.130936	0.550758	0.286529
	0.273354	0.621942	0.449263

	0.374544	0.544987	0.289629
	0.552669	0.621816	0.449228
	0.623934	0.550687	0.286523
	0.796708	0.629086	0.443729
	0.874243	0.544305	0.273545
	0.042489	0.880442	0.441689
	0.120311	0.800393	0.275844
	0.289416	0.885184	0.441569
	0.374532	0.800011	0.289626
	0.552734	0.901509	0.449014
	0.629753	0.800148	0.289591
	0.799897	0.885226	0.4415
	0.884039	0.800272	0.27583
	0.459686	0.715183	0.498776

Ta@VS_2			
	1		
	12.884	0	0
	-6.442	11.15787	0
	0	0	17.8775
	V	S	Ta
	16	32	1
Direct			
	0.201992	0.200306	0.362145
	0.47793	0.224166	0.35741
	0.713006	0.221605	0.358723
	0.950565	0.224291	0.357467
	0.187857	0.433329	0.359216
	0.449584	0.433263	0.359141
	0.712764	0.461863	0.358649
	0.953213	0.462021	0.35866
	0.187635	0.724739	0.359137
	0.459719	0.71507	0.359004
	0.741207	0.72476	0.359318
	0.950364	0.696781	0.357575
	0.202608	0.97291	0.362275
	0.449695	0.986824	0.35927
	0.741349	0.986992	0.35924
	0.97445	0.9722	0.362304
	0.042292	0.132413	0.441423
	0.126191	0.048255	0.274036
	0.294359	0.132622	0.441417
	0.374551	0.054626	0.275373
	0.545223	0.138296	0.44376
	0.62381	0.043376	0.28689
	0.797343	0.138363	0.443824
	0.884221	0.05461	0.275436
	0.036363	0.377517	0.443791
	0.119956	0.290386	0.275291
	0.289091	0.373992	0.441139
	0.37451	0.290372	0.275315
	0.54521	0.377436	0.443746
	0.630868	0.300742	0.273544
	0.792959	0.381776	0.442713
	0.874048	0.300762	0.273569
	0.036334	0.629453	0.443859
	0.131355	0.550908	0.286823
	0.272302	0.621369	0.450989

	0.374158	0.544105	0.289726
	0.553259	0.621415	0.450929
	0.623727	0.550916	0.286849
	0.797168	0.629337	0.443906
	0.874075	0.544022	0.273584
	0.042436	0.880448	0.441467
	0.119977	0.800119	0.275476
	0.289122	0.885537	0.441114
	0.374176	0.800429	0.289796
	0.553257	0.902436	0.450981
	0.630519	0.800568	0.289785
	0.800574	0.885547	0.441194
	0.884245	0.800136	0.275486
	0.459591	0.715061	0.497987

Y@VS_2			
	1		
	12.884	0	0
	-6.442	11.15787	0
	0	0	17.8775
	V	S	Y
	16	32	1
Direct			
	0.192744	0.180995	0.363144
	0.471056	0.210336	0.360247
	0.710179	0.216396	0.361034
	0.943363	0.210448	0.360249
	0.177942	0.449431	0.356356
	0.475606	0.449508	0.356397
	0.715804	0.455434	0.360393
	0.943514	0.455239	0.360399
	0.191371	0.730646	0.363249
	0.475095	0.746364	0.356053
	0.743383	0.730504	0.363164
	0.960375	0.716338	0.358621
	0.206499	0.962724	0.358529
	0.470905	0.981949	0.360391
	0.715175	0.981856	0.360407
	0.960554	0.962866	0.358532
	0.036537	0.125069	0.441846
	0.123355	0.042431	0.272836
	0.292619	0.124901	0.441918
	0.38	0.050468	0.277447
	0.54574	0.133407	0.446727
	0.628559	0.052866	0.276617
	0.791748	0.133335	0.446699
	0.874702	0.050548	0.277494
	0.03423	0.377643	0.445043
	0.122803	0.291745	0.282939
	0.283843	0.36335	0.438616
	0.373075	0.291761	0.282869
	0.547529	0.377597	0.445009
	0.628648	0.296977	0.276428
	0.791733	0.37946	0.446864
	0.872379	0.29692	0.276363
	0.036374	0.63178	0.442816
	0.122647	0.551669	0.282583
	0.283718	0.640544	0.438601

	0.376214	0.548406	0.301308
	0.561022	0.640631	0.438649
	0.633182	0.551714	0.282526
	0.799612	0.631836	0.442781
	0.874578	0.544944	0.276938
	0.042949	0.881568	0.439581
	0.123081	0.800784	0.2728
	0.292914	0.887475	0.441825
	0.372973	0.802152	0.282959
	0.547266	0.890355	0.444853
	0.633206	0.802037	0.283037
	0.798816	0.88736	0.441774
	0.881932	0.800828	0.272792
	0.376182	0.548115	0.512047

Zr@VS_2			
	1		
	12.884	0	0
	-6.442	11.15787	0
	0	0	17.8775
	V	S	Zr
	16	32	1
Direct			
	0.226444	0.214782	0.360956
	0.467757	0.212299	0.363306
	0.717586	0.231353	0.361589
	0.934422	0.188739	0.353562
	0.230174	0.449278	0.355613
	0.465473	0.457345	0.360765
	0.694679	0.451676	0.363375
	0.959102	0.488624	0.367395
	0.192286	0.722577	0.367752
	0.492409	0.742827	0.354205
	0.722486	0.704335	0.362863
	0.964412	0.71612	0.365546
	0.227584	0.983877	0.364044
	0.44635	0.958966	0.362102
	0.699767	0.980199	0.354226
	0.974723	0.958129	0.364307
	0.042837	0.132958	0.43525
	0.134268	0.04967	0.279139
	0.301184	0.136279	0.447906
	0.379009	0.048426	0.278387
	0.547688	0.131026	0.442483
	0.621784	0.055396	0.276198
	0.79062	0.129927	0.442204
	0.872019	0.043392	0.273801
	0.039714	0.36992	0.43994
	0.126971	0.292187	0.282329
	0.301724	0.379152	0.445259
	0.383535	0.296968	0.278293
	0.543267	0.378758	0.447403
	0.629452	0.29965	0.277548
	0.791333	0.38317	0.44781
	0.886266	0.311606	0.285444
	0.033218	0.646801	0.457101
	0.131028	0.549098	0.294997
	0.309875	0.639994	0.439565

	0.387197	0.553367	0.281647
	0.549155	0.637536	0.43655
	0.63065	0.546021	0.278076
	0.789451	0.628426	0.446988
	0.878205	0.553772	0.282986
	0.050734	0.89035	0.44767
	0.126636	0.802462	0.282764
	0.295884	0.887816	0.449105
	0.368111	0.791941	0.286403
	0.549867	0.888432	0.442149
	0.637574	0.807336	0.274107
	0.795468	0.884342	0.429736
	0.882584	0.799229	0.279363
	0.131589	0.548581	0.504262

Pd@VS_2			
	1		
	12.884	0	0
	-6.442	11.15787	0
	0	0	17.8775
	V	S	Pd
	16	32	1
Direct			
	0.205426	0.206763	0.3609
	0.449561	0.210208	0.360114
	0.705173	0.205631	0.358894
	0.964673	0.210121	0.360108
	0.206986	0.449246	0.361238
	0.446263	0.449282	0.361254
	0.705247	0.470086	0.358794
	0.969235	0.469905	0.358831
	0.206968	0.72834	0.361237
	0.459529	0.714854	0.363346
	0.725529	0.728395	0.361219
	0.964613	0.725135	0.360116
	0.205333	0.969222	0.360829
	0.446289	0.967612	0.361155
	0.725426	0.967456	0.36122
	0.968082	0.96934	0.360873
	0.044198	0.130479	0.444904
	0.126316	0.048431	0.275191
	0.290386	0.130424	0.444895
	0.373013	0.048583	0.276704
	0.540964	0.133141	0.441955
	0.623269	0.042313	0.282379
	0.796347	0.133124	0.442128
	0.879668	0.048463	0.276742
	0.041538	0.378447	0.442107
	0.126237	0.295029	0.276719
	0.290275	0.376421	0.445191
	0.373065	0.295154	0.276685
	0.541078	0.378479	0.442047
	0.624634	0.29756	0.281983
	0.792949	0.381719	0.436327
	0.877091	0.297476	0.281897
	0.041431	0.633639	0.44203
	0.132418	0.551477	0.28234
	0.28087	0.625692	0.439901

	0.375779	0.547393	0.281808
	0.54885	0.625631	0.439883
	0.623261	0.551536	0.282382
	0.796347	0.633735	0.442081
	0.877171	0.549994	0.28182
	0.04423	0.884244	0.444908
	0.126087	0.801588	0.27672
	0.290274	0.884355	0.445166
	0.375672	0.798736	0.281856
	0.548905	0.893604	0.439766
	0.627131	0.798856	0.281875
	0.798265	0.884371	0.445196
	0.879604	0.801603	0.276698
	0.459553	0.715178	0.508342

Rh@VS_2			
	1		
	12.884	0	0
	-6.442	11.15787	0
	0	0	17.8775
	V	S	Rh
	16	32	1
Direct			
	0.215975	0.208648	0.361938
	0.465927	0.216774	0.360883
	0.704209	0.208341	0.361686
	0.953127	0.205441	0.359844
	0.221295	0.45352	0.363407
	0.460996	0.451198	0.360684
	0.701013	0.454235	0.360834
	0.94414	0.455861	0.367068
	0.222466	0.73839	0.360695
	0.469645	0.71915	0.364431
	0.721787	0.740625	0.359412
	0.949861	0.711406	0.364534
	0.227294	0.980689	0.361817
	0.463907	0.978545	0.361418
	0.701814	0.971748	0.359338
	0.979852	0.970709	0.359885
	0.047535	0.130283	0.441172
	0.135554	0.05034	0.276174
	0.299412	0.134512	0.44624
	0.381919	0.052926	0.276127
	0.545705	0.134328	0.446152
	0.625312	0.051447	0.275539
	0.7908	0.129932	0.442968
	0.879278	0.050011	0.279837
	0.034149	0.35881	0.450403
	0.130034	0.298501	0.282638
	0.298435	0.377519	0.446744
	0.379767	0.296151	0.277908
	0.544542	0.379843	0.445683
	0.628131	0.297796	0.277879
	0.787627	0.377887	0.447286
	0.879292	0.3021	0.282963
	0.031963	0.644508	0.452216
	0.130643	0.550302	0.297053
	0.324028	0.64394	0.452219

	0.379563	0.547739	0.283413
	0.553595	0.633902	0.438191
	0.628913	0.550035	0.283855
	0.787683	0.633876	0.439897
	0.878401	0.54689	0.283474
	0.05029	0.888082	0.441443
	0.129744	0.800534	0.283616
	0.299864	0.889728	0.446686
	0.379005	0.800424	0.282609
	0.548374	0.890229	0.442521
	0.627935	0.803405	0.276744
	0.799307	0.890996	0.440176
	0.882458	0.80122	0.278014
	0.13198	0.551646	0.468756

Li_2S-Pd@VS_2				
	1			
	12.884	0	0	
	-6.442	11.15787	0	
	0	0	17.8775	
	V	S	Pd	Li
	16	33	1	2
Direct				
	0.188269	0.214413	0.366229	
	0.46264	0.211651	0.363504	
	0.697669	0.196331	0.367376	
	0.949754	0.222796	0.365672	
	0.208198	0.458431	0.362084	
	0.431955	0.438976	0.359844	
	0.691911	0.480452	0.366787	
	0.977233	0.475268	0.366329	
	0.205208	0.735393	0.364571	
	0.437923	0.737271	0.363068	
	0.73599	0.741865	0.359564	
	0.962713	0.711641	0.362222	
	0.193436	0.982747	0.368236	
	0.438907	0.96937	0.36529	
	0.716228	0.96546	0.362494	
	0.961195	0.985821	0.366251	
	0.035009	0.139251	0.451379	
	0.117744	0.058352	0.280229	
	0.286043	0.140678	0.446192	
	0.365796	0.054575	0.282447	
	0.534908	0.133933	0.449586	
	0.617791	0.047882	0.283808	
	0.787073	0.132323	0.449351	
	0.87267	0.0585	0.281809	
	0.041453	0.387318	0.448882	
	0.115484	0.302006	0.28163	
	0.283607	0.381445	0.4489	
	0.362894	0.292227	0.279459	
	0.53339	0.383319	0.447012	
	0.622515	0.303895	0.28851	
	0.787566	0.386626	0.433342	
	0.867353	0.30486	0.287846	
	0.040309	0.638875	0.448143	
	0.126077	0.556354	0.282845	
	0.282643	0.637569	0.444235	

	0.369557	0.55282	0.285365	
	0.535401	0.639703	0.41699	
	0.616406	0.557479	0.285345	
	0.79196	0.640503	0.446222	
	0.869766	0.551081	0.288232	
	0.034799	0.888391	0.445729	
	0.120604	0.809558	0.281932	
	0.282389	0.891523	0.448554	
	0.364026	0.81145	0.277329	
	0.537752	0.892393	0.444744	
	0.622474	0.80511	0.285183	
	0.793888	0.890005	0.448751	
	0.882755	0.811625	0.278924	
	0.542588	0.62777	0.604805	
	0.475293	0.702907	0.515459	
	0.725469	0.728547	0.543963	
	0.442178	0.444732	0.544516	

Li_2S_2-Pd@VS_2				
	1			
	12.884	0	0	
	-6.442	11.15787	0	
	0	0	17.8775	
	V	S	Pd	Li
	16	34	1	2
Direct				
	0.194297	0.210137	0.366153	
	0.471287	0.210842	0.362231	
	0.704369	0.195517	0.364758	
	0.955996	0.222474	0.364382	
	0.212848	0.452919	0.359647	
	0.440983	0.437583	0.359514	
	0.698305	0.479628	0.367785	
	0.983991	0.475762	0.364982	
	0.207858	0.732468	0.361497	
	0.437867	0.726622	0.360129	
	0.740146	0.73731	0.358209	
	0.967291	0.708867	0.360742	
	0.199945	0.980167	0.365942	
	0.441783	0.967485	0.36218	
	0.728099	0.964985	0.357954	
	0.967699	0.979366	0.364203	
	0.039256	0.13423	0.44953	
	0.123726	0.055934	0.278554	
	0.291071	0.136275	0.445036	
	0.371433	0.053907	0.280019	
	0.540995	0.130785	0.448008	
	0.623611	0.045524	0.282003	
	0.792778	0.129036	0.446803	
	0.881608	0.057868	0.278927	
	0.047113	0.384721	0.447378	
	0.121422	0.298095	0.279973	
	0.291196	0.379132	0.447454	
	0.369395	0.290182	0.279175	
	0.538218	0.37988	0.446205	
	0.628046	0.301816	0.286091	
	0.793329	0.384412	0.432068	
	0.874057	0.304582	0.286485	
	0.044829	0.63709	0.447087	
	0.131246	0.55302	0.280988	
	0.282876	0.631509	0.44037	

	0.374687	0.548086	0.282326	
	0.541544	0.636425	0.418462	
	0.62265	0.553575	0.284053	
	0.796276	0.640002	0.445351	
	0.873914	0.547403	0.286986	
	0.040047	0.885292	0.444238	
	0.124324	0.806082	0.279351	
	0.28746	0.887897	0.446491	
	0.368258	0.807593	0.275183	
	0.545483	0.89077	0.437788	
	0.627567	0.802227	0.283141	
	0.797363	0.885279	0.445917	
	0.886641	0.806984	0.276915	
	0.505546	0.641359	0.630264	
	0.416379	0.738029	0.634839	
	0.474193	0.702006	0.514596	
	0.550135	0.924825	0.568609	
	0.329843	0.481676	0.566553	

Li_2S_4-Pd@VS_2				
	1			
	12.884	0	0	
	-6.442	11.15787	0	
	0	0	17.8775	
	V	S	Pd	Li
	16	36	1	2
Direct				
	0.203137	0.215921	0.368947	
	0.467894	0.21197	0.364649	
	0.715473	0.244626	0.370314	
	0.943688	0.226361	0.369227	
	0.211986	0.453131	0.365485	
	0.440425	0.44673	0.365358	
	0.698922	0.481129	0.365625	
	0.980318	0.475562	0.361859	
	0.213208	0.746797	0.370279	
	0.444049	0.730115	0.369224	
	0.739382	0.734684	0.362787	
	0.96295	0.706375	0.362349	
	0.191929	0.979966	0.369236	
	0.47984	0.981997	0.36348	
	0.714614	0.96062	0.363708	
	0.962544	0.992833	0.368551	
	0.040673	0.144641	0.451769	
	0.122381	0.059798	0.282337	
	0.293596	0.139912	0.44821	
	0.377522	0.055792	0.287167	
	0.546174	0.139364	0.45063	
	0.631095	0.057808	0.28538	
	0.789689	0.142074	0.447911	
	0.876296	0.061962	0.283791	
	0.045207	0.390634	0.449276	
	0.122769	0.302546	0.285377	
	0.291087	0.383544	0.453231	
	0.372452	0.298681	0.283657	
	0.540642	0.386276	0.448268	
	0.627548	0.312608	0.284601	
	0.79487	0.39659	0.44741	
	0.872294	0.308599	0.283597	
	0.044338	0.636607	0.448277	
	0.133924	0.557623	0.286254	
	0.283802	0.636981	0.445503	

	0.375963	0.556879	0.28851	
	0.546595	0.640947	0.43399	
	0.620931	0.561542	0.287464	
	0.800844	0.642282	0.446331	
	0.876317	0.552998	0.28382	
	0.039103	0.889842	0.446421	
	0.12772	0.812863	0.283675	
	0.292642	0.896688	0.448507	
	0.373378	0.812273	0.28425	
	0.551905	0.899558	0.449917	
	0.625735	0.803729	0.287455	
	0.798251	0.891491	0.449427	
	0.886736	0.812258	0.282642	
	0.730955	0.623441	0.603328	
	0.691498	0.653837	0.710252	
	0.443571	0.629357	0.635717	
	0.596212	0.74231	0.696455	
	0.465969	0.718687	0.52079	
	0.703955	0.784788	0.564192	
	0.525224	0.517134	0.552756	

Li_2S_6-Pd@VS_2				
	1			
	12.884	0	0	
	-6.442	11.15787	0	
	0	0	17.8775	
	V	S	Pd	Li
	16	38	1	2
Direct				
	0.198154	0.208609	0.376272	
	0.465076	0.219222	0.371824	
	0.695698	0.212126	0.37073	
	0.936318	0.223755	0.369456	
	0.189697	0.442466	0.37062	
	0.440351	0.447213	0.367503	
	0.698399	0.45424	0.372118	
	0.966771	0.487487	0.370667	
	0.208691	0.747004	0.368517	
	0.465538	0.748802	0.370059	
	0.719111	0.755215	0.368141	
	0.938883	0.70702	0.375008	
	0.222014	0.99024	0.372963	
	0.451026	0.975268	0.372195	
	0.705056	0.985223	0.370154	
	0.971733	0.969997	0.375846	
	0.036127	0.137256	0.453071	
	0.124359	0.053843	0.289194	
	0.29352	0.142	0.457045	
	0.374483	0.057836	0.286976	
	0.539133	0.140296	0.456507	
	0.619195	0.056692	0.286839	
	0.787054	0.144984	0.456177	
	0.874154	0.059276	0.294079	
	0.037401	0.390009	0.453576	
	0.115122	0.295307	0.288661	
	0.285692	0.383975	0.455702	
	0.367421	0.297204	0.288267	
	0.536833	0.385242	0.451683	
	0.621149	0.30289	0.286444	
	0.789633	0.389186	0.451562	
	0.868128	0.309829	0.290405	
	0.042488	0.644618	0.453652	
	0.128048	0.559058	0.29432	
	0.284341	0.636544	0.437152	

	0.37215	0.556503	0.299998	
	0.548239	0.641747	0.443493	
	0.621012	0.56045	0.301854	
	0.784706	0.638238	0.452922	
	0.86903	0.554567	0.289827	
	0.043063	0.887704	0.45371	
	0.120837	0.811339	0.290762	
	0.290163	0.895065	0.456179	
	0.373219	0.812028	0.286777	
	0.544413	0.903779	0.455958	
	0.626408	0.815493	0.28547	
	0.793418	0.899852	0.454053	
	0.877527	0.807301	0.28991	
	0.529217	0.446604	0.605744	
	0.553173	0.445214	0.720035	
	0.610892	0.718109	0.774328	
	0.687078	0.612785	0.748852	
	0.400913	0.669819	0.643663	
	0.580569	0.779152	0.674775	
	0.446286	0.713016	0.518481	
	0.365102	0.476817	0.58306	
	0.647487	0.657957	0.572275	

Li_2S_8-Pd@VS_2				
	1			
	12.884	0	0	
	-6.442	11.15787	0	
	0	0	17.8775	
	V	S	Pd	Li
	16	40	1	2
Direct				
	0.200676	0.21306	0.36609	
	0.449622	0.206852	0.362127	
	0.699243	0.198735	0.364494	
	0.962585	0.227771	0.364863	
	0.204508	0.457908	0.365828	
	0.434288	0.450593	0.363826	
	0.699023	0.484066	0.365385	
	0.970068	0.469588	0.364973	
	0.203871	0.726897	0.364583	
	0.434642	0.712923	0.365604	
	0.732808	0.739057	0.362144	
	0.963536	0.710544	0.363721	
	0.195879	0.971097	0.365542	
	0.437477	0.960968	0.363873	
	0.725851	0.970645	0.362661	
	0.966653	0.984792	0.365471	
	0.042118	0.138733	0.450083	
	0.122766	0.054989	0.280138	
	0.288189	0.133978	0.446747	
	0.367849	0.050195	0.28029	
	0.535547	0.13211	0.44526	
	0.619603	0.044111	0.283765	
	0.791132	0.134058	0.44678	
	0.878075	0.05722	0.280563	
	0.039353	0.383603	0.449984	
	0.122603	0.301487	0.282049	
	0.286034	0.381063	0.450978	
	0.369787	0.298505	0.281121	
	0.535989	0.382338	0.444242	
	0.623574	0.302754	0.289984	
	0.786626	0.383971	0.435398	
	0.872722	0.302008	0.285286	
	0.038958	0.634784	0.448411	
	0.126139	0.552039	0.284089	
	0.275371	0.62978	0.446922	

	0.368567	0.549849	0.282808	
	0.542398	0.635506	0.435531	
	0.619172	0.557887	0.284717	
	0.793351	0.640624	0.447018	
	0.871695	0.549621	0.286318	
	0.038475	0.886567	0.445971	
	0.121063	0.802588	0.281087	
	0.28392	0.885443	0.447904	
	0.366817	0.801511	0.280271	
	0.540663	0.893299	0.439456	
	0.621471	0.801011	0.288126	
	0.797528	0.890448	0.448917	
	0.881532	0.807899	0.281643	
	0.503198	0.499532	0.772556	
	0.583811	0.874159	0.783367	
	0.608284	0.446447	0.718026	
	0.602188	0.629755	0.85345	
	0.509818	0.703673	0.633589	
	0.440225	0.753102	0.721235	
	0.687464	0.550773	0.623624	
	0.690949	0.794911	0.800891	
	0.458366	0.722872	0.514894	
	0.696918	0.724184	0.672172	
	0.515693	0.499842	0.547077	

S_8-Pd@VS_2				
	1			
	12.884	0	0	
	-6.442	11.15787	0	
	0	0	17.8775	
	V	S	Pd	
	16	40	1	
Direct				
	0.207471	0.20491	0.358869	
	0.472761	0.236503	0.356712	
	0.700241	0.19228	0.360361	
	0.966415	0.217721	0.355085	
	0.216987	0.455776	0.358329	
	0.451808	0.45886	0.360386	
	0.707076	0.479913	0.358818	
	0.984166	0.480118	0.359833	
	0.207363	0.736523	0.354739	
	0.467108	0.705343	0.361507	
	0.720725	0.727025	0.359416	
	0.948282	0.712831	0.357453	
	0.19419	0.963973	0.357929	
	0.43991	0.974043	0.353858	
	0.725593	0.961468	0.356529	
	0.965483	0.968816	0.358076	
	0.043059	0.1336	0.439713	
	0.124442	0.049596	0.272474	
	0.291685	0.132229	0.438635	
	0.37236	0.056127	0.274379	
	0.538576	0.133226	0.439122	
	0.622343	0.041941	0.277	
	0.794862	0.130003	0.439598	
	0.880183	0.051792	0.273444	
	0.046771	0.382676	0.439542	
	0.127416	0.299092	0.274473	
	0.291992	0.378063	0.442645	
	0.376497	0.300759	0.275868	
	0.545105	0.384412	0.443416	
	0.62941	0.302394	0.279182	
	0.795859	0.382096	0.427617	
	0.875119	0.301156	0.283336	
	0.045011	0.638497	0.439791	
	0.134396	0.554928	0.276784	
	0.287013	0.631629	0.431772	

	0.37763	0.54679	0.276075	
	0.549708	0.628642	0.441834	
	0.625117	0.553492	0.278105	
	0.79473	0.635512	0.443569	
	0.875938	0.548642	0.279734	
	0.039924	0.884031	0.441863	
	0.120579	0.80183	0.273147	
	0.288393	0.888214	0.440738	
	0.376771	0.800483	0.280568	
	0.546527	0.8901	0.427412	
	0.628744	0.798396	0.27587	
	0.798407	0.885674	0.443032	
	0.876202	0.800604	0.274874	
	0.229046	0.524038	0.631614	
	0.303188	0.431413	0.681585	
	0.289733	0.685663	0.682568	
	0.453514	0.463927	0.621847	
	0.444476	0.811361	0.624674	
	0.605044	0.587251	0.678222	
	0.598132	0.844701	0.686708	
	0.669169	0.756037	0.632178	
	0.457996	0.728373	0.509634	

Li_2S-Nb@VS_2				
	1			
	12.884	0	0	
	-6.442	11.15787	0	
	0	0	17.8775	
	V	S	Nb	Li
	16	33	1	2
Direct				
	0.477094	0.233906	0.360855	
	0.706142	0.197313	0.360621	
	0.964317	0.233658	0.359709	
	0.191046	0.450165	0.359657	
	0.492745	0.468916	0.361792	
	0.719235	0.448986	0.364673	
	0.959211	0.465986	0.363209	
	0.205977	0.747646	0.360719	
	0.46404	0.683576	0.351481	
	0.720152	0.734017	0.359264	
	0.940173	0.692256	0.362343	
	0.208491	0.97217	0.361721	
	0.445115	0.983097	0.351383	
	0.745931	0.985562	0.348918	
	0.966176	0.948861	0.360636	
	0.039799	0.127223	0.440573	
	0.126503	0.047679	0.275626	
	0.295237	0.132612	0.444488	
	0.373823	0.060838	0.274508	
	0.542224	0.125873	0.441363	
	0.622857	0.037414	0.282018	
	0.795267	0.125697	0.441468	
	0.895077	0.058287	0.274927	
	0.040865	0.380834	0.447598	
	0.125932	0.295851	0.279072	
	0.296576	0.3796	0.435102	
	0.38117	0.300784	0.285419	
	0.556345	0.37974	0.450132	
	0.631678	0.294949	0.278889	
	0.797903	0.373774	0.446866	
	0.877185	0.299872	0.278611	
	0.047485	0.636052	0.442861	
	0.135299	0.559147	0.287012	
	0.300027	0.645052	0.431801	
	0.377567	0.528317	0.280899	

	0.563115	0.637453	0.442454	
	0.64007	0.548331	0.281113	
	0.79435	0.627569	0.443789	
	0.875811	0.540434	0.277163	
	0.042473	0.87517	0.442801	
	0.123174	0.804241	0.276667	
	0.30078	0.896525	0.444918	
	0.383887	0.798398	0.295585	
	0.545901	0.880003	0.550327	
	0.630448	0.803426	0.278817	
	0.800112	0.890652	0.440823	
	0.88014	0.793409	0.275905	
	0.376824	0.551276	0.615563	
	0.450668	0.690372	0.522383	
	0.443174	0.440087	0.534149	
	0.210576	0.451757	0.52832	

Li_2S_2-Nb@VS_2				
	1			
	12.884	0	0	
	-6.442	11.15787	0	
	0	0	17.8775	
	V	S	Nb	Li
	16	34	1	2
Direct				
	0.182484	0.202514	0.364989	
	0.471309	0.225241	0.363513	
	0.712959	0.221757	0.365247	
	0.95633	0.233781	0.364141	
	0.204025	0.442367	0.363446	
	0.441478	0.437052	0.359056	
	0.693799	0.465302	0.362979	
	0.936867	0.464154	0.363736	
	0.173282	0.705928	0.360996	
	0.415292	0.680581	0.345973	
	0.736373	0.729617	0.360363	
	0.944584	0.698731	0.362751	
	0.205591	0.982075	0.366961	
	0.448678	0.992487	0.364868	
	0.733561	0.969914	0.352707	
	0.965511	0.956385	0.363962	
	0.029695	0.126767	0.444252	
	0.122219	0.048176	0.277881	
	0.285687	0.134758	0.445324	
	0.37151	0.057328	0.279654	
	0.538896	0.138518	0.450084	
	0.619035	0.042857	0.292122	
	0.788236	0.126687	0.442801	
	0.881737	0.051195	0.277759	
	0.032866	0.380194	0.446483	
	0.113304	0.290415	0.277684	
	0.283842	0.371088	0.447482	
	0.36735	0.288401	0.279288	
	0.533664	0.37889	0.448667	
	0.6238	0.300787	0.280698	
	0.781687	0.380274	0.447976	
	0.868989	0.301508	0.279511	
	0.027105	0.626317	0.449236	
	0.115026	0.542117	0.289359	
	0.27313	0.613796	0.441913	

	0.354044	0.523564	0.272762	
	0.536555	0.635367	0.425477	
	0.616579	0.551211	0.285528	
	0.784857	0.63085	0.448584	
	0.862784	0.544562	0.278971	
	0.041158	0.879363	0.44593	
	0.113932	0.796261	0.281785	
	0.284374	0.8807	0.440225	
	0.367879	0.804441	0.290726	
	0.536083	0.897506	0.459025	
	0.614182	0.793024	0.291944	
	0.796354	0.883564	0.444183	
	0.880336	0.798909	0.275183	
	0.430412	0.704059	0.643648	
	0.594553	0.724457	0.607865	
	0.455234	0.716036	0.510738	
	0.600139	0.555076	0.529537	
	0.713304	0.957903	0.535135	

Li_2S_4-Nb@VS_2				
	1			
	12.884	0	0	
	-6.442	11.15787	0	
	0	0	17.8775	
	V	S	Nb	Li
	16	36	1	2
Direct				
	0.183939	0.184449	0.365838	
	0.470904	0.226826	0.362714	
	0.704885	0.1997	0.364246	
	0.954453	0.225083	0.363417	
	0.182117	0.443287	0.363769	
	0.483686	0.464068	0.364463	
	0.710621	0.444061	0.366135	
	0.951772	0.462668	0.365246	
	0.195357	0.737286	0.362949	
	0.454921	0.679892	0.36019	
	0.711858	0.726567	0.363321	
	0.932429	0.687995	0.366317	
	0.203741	0.965362	0.363693	
	0.439109	0.97454	0.361599	
	0.73281	0.980126	0.360133	
	0.956995	0.941568	0.365124	
	0.032501	0.121243	0.444225	
	0.115613	0.038761	0.278176	
	0.285823	0.125158	0.445192	
	0.368778	0.051042	0.279261	
	0.537347	0.126504	0.446198	
	0.617067	0.035716	0.287134	
	0.791365	0.128082	0.44823	
	0.879131	0.047932	0.281154	
	0.034408	0.375267	0.450102	
	0.116507	0.28979	0.281088	
	0.290587	0.377122	0.43418	
	0.372489	0.294497	0.288415	
	0.549499	0.374865	0.451347	
	0.624583	0.289811	0.280367	
	0.791769	0.371694	0.449661	
	0.869876	0.293955	0.281463	
	0.037868	0.628291	0.44667	
	0.125449	0.551431	0.288879	
	0.282835	0.630222	0.436044	

	0.371657	0.529103	0.283422	
	0.553731	0.626906	0.447845	
	0.631891	0.544042	0.28357	
	0.785188	0.620092	0.448439	
	0.868976	0.536928	0.279726	
	0.035688	0.869436	0.446177	
	0.113976	0.797486	0.279629	
	0.286621	0.884549	0.449448	
	0.371859	0.79151	0.297271	
	0.541459	0.884496	0.447925	
	0.621394	0.797327	0.284874	
	0.793487	0.881733	0.446733	
	0.870475	0.787312	0.279917	
	0.416916	0.427513	0.631054	
	0.320776	0.492719	0.579778	
	0.547272	0.746048	0.640924	
	0.364086	0.661599	0.64396	
	0.454771	0.712574	0.515329	
	0.598123	0.572547	0.571512	
	0.359831	0.29239	0.526896	

Li_2S_6-Nb@VS_2				
	1			
	12.884	0	0	
	-6.442	11.15787	0	
	0	0	17.8775	
	V	S	Nb	Li
	16	38	1	2
Direct				
	0.179933	0.183309	0.363395	
	0.439671	0.204835	0.360196	
	0.67932	0.211422	0.361316	
	0.915869	0.208662	0.358848	
	0.175465	0.41539	0.349529	
	0.419708	0.411267	0.36048	
	0.676556	0.443738	0.360333	
	0.916559	0.454011	0.360107	
	0.162655	0.716443	0.351111	
	0.476652	0.727553	0.353998	
	0.703352	0.697367	0.356628	
	0.916951	0.685175	0.358848	
	0.182903	0.964382	0.364598	
	0.415728	0.975113	0.364209	
	0.69908	0.981572	0.356749	
	0.949568	0.953365	0.365009	
	0.012307	0.11796	0.439247	
	0.097565	0.031193	0.275985	
	0.265441	0.117101	0.447833	
	0.342373	0.039108	0.275319	
	0.515655	0.124459	0.447661	
	0.589775	0.03657	0.283776	
	0.767974	0.128237	0.445132	
	0.854544	0.043904	0.278406	
	0.023923	0.376244	0.435035	
	0.090523	0.267299	0.274316	
	0.259735	0.359882	0.441925	
	0.342704	0.271065	0.274204	
	0.514585	0.360805	0.447082	
	0.596607	0.284777	0.275629	
	0.764426	0.369072	0.445957	
	0.843886	0.290356	0.276019	
	0.016953	0.619261	0.440816	
	0.104709	0.536247	0.292252	
	0.367069	0.679999	0.584304	

	0.351799	0.539551	0.294257	
	0.512583	0.607143	0.42781	
	0.589843	0.52873	0.28237	
	0.766156	0.613013	0.444865	
	0.841071	0.531576	0.274508	
	0.014843	0.863782	0.444651	
	0.089369	0.79151	0.275422	
	0.257617	0.863554	0.443006	
	0.347989	0.783106	0.297135	
	0.516698	0.881886	0.43227	
	0.624784	0.801252	0.276617	
	0.762205	0.867738	0.435782	
	0.854869	0.785098	0.275272	
	0.625942	0.642143	0.589626	
	0.625123	0.659226	0.70639	
	0.835513	0.942629	0.734177	
	0.801631	0.769063	0.739963	
	0.653038	0.938089	0.601153	
	0.828932	0.979199	0.621851	
	0.535947	0.751028	0.521877	
	0.524535	0.777721	0.684317	
	0.831627	0.797811	0.540114	

Li_2S_8-Nb@VS_2				
	1			
	12.884	0	0	
	-6.442	11.15787	0	
	0	0	17.8775	
	V	S	Nb	Li
	16	40	1	2
Direct				
	0.186142	0.201475	0.35911	
	0.474013	0.213471	0.361538	
	0.72616	0.235073	0.361923	
	0.947926	0.219957	0.359473	
	0.191295	0.438073	0.358606	
	0.433517	0.433252	0.355248	
	0.700422	0.457597	0.363015	
	0.965194	0.476294	0.3621	
	0.186548	0.725846	0.358447	
	0.482099	0.750005	0.352625	
	0.731573	0.727001	0.367773	
	0.941153	0.699745	0.363199	
	0.219765	0.984485	0.360871	
	0.436345	0.959443	0.359326	
	0.718734	0.993101	0.358307	
	0.971312	0.960513	0.363321	
	0.034715	0.127351	0.44201	
	0.12088	0.043635	0.274652	
	0.292067	0.136532	0.44052	
	0.375103	0.052431	0.274952	
	0.543364	0.132392	0.441979	
	0.624235	0.057564	0.283757	
	0.794108	0.142069	0.446321	
	0.875528	0.052608	0.28043	
	0.041416	0.383785	0.443826	
	0.115769	0.291671	0.273697	
	0.28061	0.368254	0.439708	
	0.367404	0.28781	0.275593	
	0.53469	0.377846	0.442033	
	0.62911	0.301761	0.277387	
	0.793467	0.387629	0.446311	
	0.878928	0.308036	0.277029	
	0.038036	0.635251	0.446796	
	0.128609	0.553581	0.2843	
	0.290768	0.638844	0.433573	

	0.37481	0.552947	0.292081	
	0.537981	0.631167	0.434327	
	0.619482	0.551194	0.290514	
	0.788036	0.629459	0.449967	
	0.870355	0.547361	0.280152	
	0.0447	0.88257	0.44258	
	0.114291	0.799942	0.278157	
	0.284435	0.885414	0.443897	
	0.367408	0.799159	0.278191	
	0.538497	0.893072	0.44099	
	0.636383	0.810452	0.278358	
	0.794372	0.89017	0.439364	
	0.874832	0.796104	0.279098	
	0.341496	0.63938	0.640917	
	0.402672	0.364802	0.713541	
	0.548559	0.885256	0.623247	
	0.304691	0.493211	0.574882	
	0.648387	0.632798	0.739824	
	0.488203	0.510749	0.786033	
	0.651831	0.819823	0.580356	
	0.425035	0.427954	0.607845	
	0.451923	0.71224	0.524221	
	0.630612	0.610962	0.60419	
	0.538666	0.730826	0.708569	

S_8-Nb@VS_2			
	1		
	12.884	0	0
	-6.442	11.15787	0
	0	0	17.8775
	V	S	Nb
	16	40	1
Direct			
	0.186042	0.197308	0.356852
	0.475321	0.213948	0.355401
	0.721986	0.223364	0.356679
	0.950499	0.21938	0.354216
	0.185607	0.427096	0.354184
	0.432854	0.429699	0.348036
	0.703702	0.455653	0.355205
	0.959622	0.464584	0.355488
	0.188878	0.721607	0.353863
	0.462557	0.747544	0.359272
	0.737623	0.72209	0.35719
	0.945805	0.691544	0.354659
	0.214978	0.980179	0.358786
	0.439018	0.966422	0.35926
	0.732921	0.983746	0.352988
	0.971186	0.955749	0.358738
	0.034004	0.122452	0.438125
	0.123611	0.042101	0.270669
	0.291962	0.13446	0.436993
	0.374885	0.050679	0.272604
	0.544057	0.136342	0.440172
	0.623026	0.045249	0.284522
	0.796574	0.135978	0.440747
	0.880532	0.048652	0.273141
	0.037991	0.376169	0.440318
	0.114957	0.28438	0.268173
	0.283484	0.368195	0.437472
	0.366043	0.283312	0.270383
	0.536545	0.378632	0.435411
	0.62844	0.294875	0.27143
	0.792312	0.382113	0.439088
	0.875062	0.298365	0.270515
	0.036487	0.626429	0.440283
	0.129659	0.547828	0.280065
	0.281924	0.622107	0.426866

	0.37343	0.549726	0.290042
	0.54535	0.627927	0.432786
	0.619924	0.546759	0.28254
	0.791575	0.6247	0.440831
	0.872034	0.540003	0.27138
	0.044258	0.875836	0.437652
	0.117172	0.796469	0.273223
	0.284099	0.880495	0.441527
	0.36784	0.804522	0.276547
	0.549437	0.903419	0.44398
	0.628079	0.798294	0.285312
	0.797242	0.881703	0.436862
	0.880511	0.793755	0.271208
	0.289981	0.520259	0.591487
	0.376208	0.449183	0.656929
	0.266403	0.640263	0.658617
	0.543062	0.515809	0.611396
	0.393525	0.806725	0.617497
	0.661878	0.676386	0.659076
	0.559894	0.874315	0.669148
	0.648782	0.812574	0.600841
	0.454359	0.706689	0.500376

Li_2S-Zr@VS_2				
	1			
	12.884	0	0	
	-6.442	11.15787	0	
	0	0	17.8775	
	V	S	Zr	Li
	16	33	1	2
Direct				
	0.240354	0.226277	0.361841	
	0.471924	0.213195	0.363982	
	0.71927	0.23335	0.362625	
	0.934808	0.188188	0.357474	
	0.233104	0.453791	0.358105	
	0.474082	0.46714	0.363467	
	0.697871	0.453896	0.364914	
	0.958287	0.492386	0.367359	
	0.19464	0.729505	0.368104	
	0.49238	0.746316	0.35627	
	0.719879	0.699681	0.365032	
	0.963885	0.720109	0.368124	
	0.22996	0.988199	0.363671	
	0.453471	0.962271	0.363021	
	0.70317	0.981822	0.356473	
	0.954813	0.943756	0.36729	
	0.04654	0.133109	0.434879	
	0.132206	0.054245	0.28605	
	0.306448	0.140038	0.448568	
	0.382456	0.052103	0.279747	
	0.551059	0.134267	0.444403	
	0.625149	0.057112	0.278131	
	0.791311	0.131619	0.444847	
	0.873157	0.04321	0.276178	
	0.040115	0.374334	0.436342	
	0.131145	0.295784	0.285686	
	0.308162	0.38342	0.447471	
	0.390086	0.302366	0.278709	
	0.547587	0.381612	0.448975	
	0.633263	0.302765	0.277927	
	0.793964	0.38577	0.449004	
	0.888727	0.312038	0.287393	
	0.038258	0.646682	0.45762	
	0.132722	0.552481	0.297557	
	0.308936	0.64466	0.438265	

	0.390931	0.557029	0.283801	
	0.552525	0.640825	0.441425	
	0.63372	0.547614	0.278176	
	0.792295	0.628838	0.449403	
	0.87936	0.556542	0.2828	
	0.049692	0.890399	0.448199	
	0.127507	0.80722	0.283559	
	0.297992	0.892206	0.4487	
	0.37037	0.794817	0.2874	
	0.551259	0.891699	0.444458	
	0.638338	0.80793	0.277122	
	0.792917	0.8826	0.437751	
	0.879583	0.796863	0.279757	
	0.205389	0.473174	0.615176	
	0.137453	0.542933	0.515209	
	0.388034	0.558707	0.536594	
	0.126288	0.290587	0.534565	

Li_2S_2-Zr@VS_2				
	1			
	12.884	0	0	
	-6.442	11.15787	0	
	0	0	17.8775	
	V	S	Zr	Li
	16	34	1	2
Direct				
	0.241657	0.228791	0.362849	
	0.468872	0.208129	0.363407	
	0.70656	0.214679	0.361849	
	0.932607	0.187908	0.357218	
	0.23327	0.454517	0.359301	
	0.474607	0.471362	0.363465	
	0.694024	0.446603	0.362667	
	0.958901	0.492416	0.365481	
	0.201044	0.736182	0.367415	
	0.493888	0.749594	0.354176	
	0.721397	0.700149	0.362415	
	0.970539	0.725811	0.363996	
	0.228761	0.988536	0.361582	
	0.46437	0.966896	0.361763	
	0.707095	0.979028	0.356108	
	0.948632	0.942132	0.363646	
	0.0447	0.131031	0.432747	
	0.132737	0.056993	0.285658	
	0.304786	0.139244	0.448547	
	0.383085	0.051804	0.278575	
	0.548334	0.132778	0.447039	
	0.626135	0.053664	0.275123	
	0.79	0.128169	0.445432	
	0.874385	0.044015	0.274262	
	0.038873	0.373657	0.43723	
	0.131237	0.297127	0.286608	
	0.309355	0.386226	0.449252	
	0.389129	0.302696	0.279487	
	0.546946	0.380484	0.447335	
	0.629263	0.295741	0.276836	
	0.793732	0.382402	0.444581	
	0.885428	0.31025	0.288901	
	0.039237	0.649995	0.454523	
	0.134121	0.552657	0.299059	
	0.310704	0.648393	0.4364	

	0.390875	0.559241	0.284022	
	0.555947	0.643445	0.439589	
	0.632903	0.545411	0.277033	
	0.793771	0.627446	0.444418	
	0.879246	0.555145	0.281341	
	0.048233	0.891514	0.444723	
	0.131655	0.809675	0.279805	
	0.300176	0.894561	0.447069	
	0.372827	0.798463	0.284998	
	0.553286	0.892581	0.444581	
	0.639027	0.807608	0.274685	
	0.792321	0.879814	0.438273	
	0.879778	0.796617	0.276095	
	0.136146	0.546715	0.651124	
	0.229288	0.464517	0.607256	
	0.135905	0.548301	0.513945	
	0.11881	0.279379	0.529123	
	0.408757	0.593478	0.534657	

Li_2S_4-Zr@VS_2				
	1			
	12.884	0	0	
	-6.442	11.15787	0	
	0	0	17.8775	
	V	S	Zr	Li
	16	36	1	2
Direct				
	0.21788	0.206458	0.358407	
	0.464652	0.207217	0.361744	
	0.721515	0.230559	0.361057	
	0.943685	0.18535	0.360369	
	0.228135	0.444748	0.353645	
	0.460811	0.440867	0.358084	
	0.698444	0.450749	0.36172	
	0.954051	0.482261	0.364061	
	0.191972	0.719511	0.363019	
	0.492717	0.744555	0.356835	
	0.737856	0.723182	0.365832	
	0.967439	0.722264	0.363996	
	0.223685	0.977313	0.361887	
	0.447519	0.962653	0.361555	
	0.702502	0.977661	0.355835	
	0.981698	0.962249	0.360897	
	0.046581	0.126651	0.440433	
	0.133554	0.045275	0.274403	
	0.299027	0.130362	0.445811	
	0.378919	0.046719	0.275678	
	0.547123	0.128849	0.442199	
	0.626678	0.054242	0.277456	
	0.791549	0.129053	0.443631	
	0.875512	0.040712	0.277174	
	0.042756	0.372337	0.433453	
	0.125525	0.287157	0.278554	
	0.298221	0.371044	0.440203	
	0.381236	0.289007	0.274592	
	0.5436	0.373741	0.446089	
	0.629338	0.29579	0.277639	
	0.79317	0.381869	0.446927	
	0.886154	0.303843	0.28654	
	0.039192	0.638091	0.451662	
	0.128848	0.545643	0.291991	
	0.302016	0.63008	0.431628	

	0.38888	0.549648	0.282402	
	0.549759	0.630497	0.432031	
	0.630157	0.546842	0.282606	
	0.796507	0.630167	0.444908	
	0.875568	0.551398	0.281149	
	0.052431	0.885945	0.446677	
	0.127043	0.800324	0.278422	
	0.294274	0.882595	0.445655	
	0.372689	0.792804	0.285589	
	0.54942	0.887827	0.444029	
	0.638612	0.806336	0.275989	
	0.800157	0.885415	0.436436	
	0.884408	0.799134	0.27725	
	0.252734	0.712512	0.614913	
	0.079638	0.586228	0.650365	
	0.373189	0.492064	0.644374	
	0.210101	0.40335	0.588723	
	0.139827	0.538025	0.526904	
	0.435266	0.623937	0.540891	
	0.240211	0.550358	0.692773	

Li_2S_6-Zr@VS_2				
	1			
	12.884	0	0	
	-6.442	11.15787	0	
	0	0	17.8775	
	V	S	Zr	Li
	16	38	1	2
Direct				
	0.199427	0.199318	0.360954	
	0.473303	0.201862	0.363645	
	0.729986	0.222658	0.360593	
	0.942621	0.182829	0.355666	
	0.234988	0.447963	0.354779	
	0.458517	0.432601	0.357629	
	0.697774	0.445757	0.360658	
	0.955458	0.47847	0.364363	
	0.190428	0.714375	0.362033	
	0.493183	0.73968	0.355157	
	0.739907	0.723632	0.363267	
	0.962817	0.71249	0.363667	
	0.22779	0.976697	0.364633	
	0.449706	0.955122	0.363247	
	0.704854	0.970569	0.354499	
	0.976807	0.953286	0.362591	
	0.039888	0.121471	0.442318	
	0.132672	0.04147	0.277305	
	0.299301	0.130963	0.44471	
	0.380517	0.041973	0.277773	
	0.549857	0.124252	0.444144	
	0.625959	0.048841	0.278107	
	0.794608	0.124951	0.442671	
	0.876453	0.036489	0.274619	
	0.046097	0.373122	0.431747	
	0.12469	0.288124	0.279928	
	0.299038	0.36973	0.442443	
	0.378583	0.282562	0.278345	
	0.543439	0.369087	0.445763	
	0.631375	0.289993	0.278627	
	0.793258	0.375976	0.44557	
	0.887389	0.301461	0.282092	
	0.03524	0.633499	0.451712	
	0.13031	0.542559	0.291458	
	0.299838	0.626928	0.433322	

	0.39056	0.545219	0.280505	
	0.550243	0.627583	0.43041	
	0.62987	0.542424	0.283594	
	0.79352	0.626285	0.443736	
	0.875972	0.546163	0.27984	
	0.05026	0.881541	0.446848	
	0.127446	0.797288	0.278976	
	0.292233	0.876671	0.442953	
	0.372477	0.78802	0.284469	
	0.5534	0.885585	0.442759	
	0.639289	0.801566	0.273661	
	0.800286	0.881238	0.437949	
	0.886805	0.795947	0.275661	
	0.482073	0.772263	0.650705	
	0.373565	0.845293	0.653655	
	0.039603	0.527431	0.657997	
	0.240346	0.778171	0.576984	
	0.241199	0.460295	0.616291	
	0.056378	0.380211	0.632445	
	0.130096	0.533185	0.525747	
	0.278892	0.630688	0.693603	
	0.472358	0.704472	0.523598	

Li_2S_8-Zr@VS_2				
	1			
	12.884	0	0	
	-6.442	11.15787	0	
	0	0	17.8775	
	V	S	Zr	Li
	16	40	1	2
Direct				
	0.200599	0.224131	0.359925	
	0.479583	0.227776	0.362803	
	0.732443	0.245659	0.361314	
	0.944337	0.205824	0.354963	
	0.234926	0.4707	0.353604	
	0.457229	0.458653	0.356849	
	0.697221	0.471212	0.359694	
	0.956206	0.502771	0.363916	
	0.191771	0.73656	0.36366	
	0.497868	0.758353	0.359886	
	0.739634	0.751187	0.36285	
	0.961111	0.731183	0.363227	
	0.230921	0.000289	0.362983	
	0.448516	0.974959	0.361663	
	0.703793	0.991543	0.356408	
	0.98368	0.978179	0.360686	
	0.041623	0.142742	0.440878	
	0.137707	0.066813	0.275386	
	0.301052	0.152182	0.443665	
	0.384519	0.066445	0.277257	
	0.552234	0.147591	0.443435	
	0.628992	0.072044	0.279271	
	0.796359	0.148357	0.443395	
	0.877208	0.059707	0.275397	
	0.046763	0.39795	0.431629	
	0.125768	0.310494	0.278253	
	0.298264	0.393737	0.443515	
	0.380906	0.306649	0.278853	
	0.545894	0.394344	0.4452	
	0.635355	0.314776	0.277887	
	0.794504	0.401601	0.44505	
	0.890096	0.325484	0.282408	
	0.033807	0.658773	0.452098	
	0.131277	0.566469	0.290473	
	0.306764	0.654659	0.426954	

	0.390387	0.566924	0.278444	
	0.551535	0.651765	0.434178	
	0.630366	0.566677	0.282448	
	0.791263	0.650103	0.442746	
	0.876458	0.569439	0.27843	
	0.053959	0.904722	0.445122	
	0.128732	0.819887	0.280245	
	0.296992	0.902165	0.446894	
	0.375259	0.810701	0.285643	
	0.55244	0.906178	0.444294	
	0.640792	0.824869	0.274582	
	0.804846	0.90656	0.43853	
	0.8874	0.819554	0.275524	
	0.047315	0.497972	0.708669	
	0.115508	0.213457	0.667367	
	0.146103	0.661715	0.653572	
	-0.02581	0.379113	0.621048	
	0.419014	0.392473	0.659848	
	0.269088	0.32947	0.726676	
	0.303222	0.677209	0.613094	
	0.09262	0.315316	0.587129	
	0.140441	0.572643	0.51095	
	0.382597	0.307892	0.537212	
	0.325332	0.503822	0.639878	

S_8-Zr@VS_2			
	1		
	12.884	0	0
	-6.442	11.15787	0
	0	0	17.8775
	V	S	Zr
	16	40	1
Direct			
	0.223826	0.213904	0.352079
	0.467519	0.212397	0.355606
	0.720552	0.233576	0.354431
	0.936402	0.185421	0.347872
	0.230431	0.447768	0.347642
	0.464586	0.454862	0.352616
	0.696112	0.452607	0.356695
	0.958778	0.487397	0.359594
	0.191568	0.720732	0.359585
	0.492591	0.741944	0.349232
	0.721071	0.702046	0.356038
	0.963092	0.713869	0.357537
	0.226896	0.983679	0.355968
	0.44528	0.958797	0.354564
	0.699416	0.978963	0.348549
	0.977094	0.957616	0.355577
	0.043131	0.130182	0.429212
	0.133742	0.048498	0.269821
	0.300611	0.136032	0.439591
	0.37928	0.048799	0.270417
	0.547845	0.131578	0.434281
	0.623056	0.055572	0.270043
	0.79126	0.130127	0.435527
	0.871322	0.040697	0.267277
	0.041547	0.370738	0.424664
	0.126555	0.290902	0.27314
	0.300942	0.376259	0.436589
	0.383424	0.296059	0.269878
	0.543017	0.378217	0.439918
	0.630644	0.300382	0.270674
	0.791817	0.383271	0.441439
	0.886102	0.308866	0.278405
	0.034667	0.644778	0.447334
	0.131065	0.548993	0.2865
	0.30703	0.637845	0.428525

	0.388234	0.552926	0.274098
	0.54823	0.634996	0.430058
	0.630295	0.544961	0.270513
	0.790333	0.62827	0.440409
	0.8773	0.552145	0.274621
	0.05108	0.888905	0.439806
	0.12658	0.801548	0.274865
	0.295155	0.887926	0.441168
	0.370083	0.792587	0.27922
	0.548793	0.888267	0.436205
	0.636956	0.806306	0.26825
	0.796093	0.882889	0.421788
	0.881079	0.797044	0.271889
	0.948879	0.351161	0.603527
	0.082127	0.323753	0.647886
	0.901768	0.440468	0.682898
	0.241271	0.454332	0.598617
	0.973654	0.611881	0.642362
	0.328198	0.61369	0.660271
	0.139352	0.724389	0.687395
	0.266636	0.721659	0.614925
	0.130385	0.543834	0.499589

Li_2S-Hf@VS_2				
	1			
	12.884	0	0	
	-6.442	11.15787	0	
	0	0	17.8775	
	V	S	Hf	Li
	16	33	1	2
Direct				
	0.214526	0.21274	0.368066	
	0.485748	0.210523	0.365601	
	0.721678	0.222797	0.366431	
	0.945301	0.215551	0.364714	
	0.224594	0.440711	0.360981	
	0.458256	0.436885	0.368649	
	0.710163	0.464302	0.366658	
	0.948087	0.461902	0.364882	
	0.182289	0.713424	0.354483	
	0.492647	0.752132	0.356494	
	0.748433	0.724092	0.362679	
	0.958265	0.698421	0.360684	
	0.204188	0.977976	0.368314	
	0.492859	0.98191	0.365063	
	0.727673	0.971113	0.365396	
	0.972375	0.986752	0.365633	
	0.045183	0.135436	0.448432	
	0.132947	0.052435	0.280343	
	0.300471	0.127361	0.44828	
	0.38529	0.046401	0.289187	
	0.556609	0.132057	0.452225	
	0.642247	0.056129	0.281974	
	0.798947	0.134492	0.450506	
	0.88707	0.056865	0.279866	
	0.050588	0.385431	0.440636	
	0.132304	0.291271	0.281433	
	0.295434	0.364626	0.455666	
	0.38498	0.29002	0.285306	
	0.551316	0.374347	0.451856	
	0.636127	0.300628	0.282587	
	0.797989	0.382443	0.450846	
	0.881082	0.303241	0.27954	
	0.043512	0.630359	0.447671	
	0.132939	0.540802	0.290374	
	0.299528	0.637984	0.42309	

	0.392452	0.551589	0.292982	
	0.563088	0.639322	0.441004	
	0.628459	0.549256	0.290565	
	0.804496	0.63182	0.448701	
	0.873799	0.543286	0.278615	
	0.04507	0.881708	0.441901	
	0.122984	0.801341	0.275717	
	0.295926	0.877521	0.435537	
	0.382896	0.805185	0.28666	
	0.559387	0.88905	0.448966	
	0.64929	0.806461	0.281633	
	0.806961	0.885001	0.445837	
	0.891271	0.802993	0.279873	
	0.275863	0.56721	0.616847	
	0.378218	0.560668	0.509172	
	0.10769	0.394292	0.566731	
	0.229695	0.702049	0.531701	

Li_2S_2-Hf@VS_2				
	1			
	12.884	0	0	
	-6.442	11.15787	0	
	0	0	17.8775	
	V	S	Hf	Li
	16	34	1	2
Direct				
	0.204857	0.182425	0.355365	
	0.446875	0.185134	0.362295	
	0.70349	0.215702	0.362192	
	0.952286	0.209362	0.361805	
	0.176136	0.452104	0.358181	
	0.485362	0.496064	0.362558	
	0.726427	0.459804	0.359698	
	0.95151	0.444754	0.360121	
	0.194232	0.731289	0.362981	
	0.488007	0.736457	0.361594	
	0.718259	0.712373	0.363156	
	0.964534	0.739442	0.362124	
	0.215086	0.962068	0.359772	
	0.465491	0.959719	0.360905	
	0.711204	0.987908	0.361812	
	0.936743	0.964153	0.360343	
	0.043446	0.130061	0.43963	
	0.122265	0.039026	0.274694	
	0.287354	0.119123	0.446028	
	0.37534	0.04032	0.275403	
	0.539806	0.126875	0.445834	
	0.623079	0.051357	0.277242	
	0.79254	0.136419	0.447015	
	0.870303	0.051587	0.275429	
	0.035258	0.375793	0.447542	
	0.125408	0.291984	0.283557	
	0.288623	0.374188	0.434135	
	0.380445	0.30346	0.293496	
	0.542685	0.378556	0.437898	
	0.618879	0.29604	0.282965	
	0.793947	0.378039	0.446382	
	0.86902	0.293428	0.277596	
	0.037641	0.632459	0.438312	
	0.119477	0.547418	0.280009	
	0.285414	0.637193	0.43917	

	0.37344	0.550201	0.292775	
	0.564697	0.651615	0.450114	
	0.635072	0.553024	0.279624	
	0.802672	0.635164	0.443561	
	0.878999	0.547373	0.28163	
	0.041949	0.889866	0.441407	
	0.122314	0.79996	0.274203	
	0.290749	0.882196	0.445078	
	0.373628	0.791618	0.286254	
	0.549597	0.890266	0.44722	
	0.634112	0.806784	0.280552	
	0.793624	0.890361	0.443671	
	0.875576	0.80284	0.275693	
	0.453877	0.455533	0.612363	
	0.340101	0.521668	0.646004	
	0.37214	0.544587	0.512422	
	0.636165	0.536023	0.531381	
	0.365038	0.289257	0.527588	

Li_2S_4-Hf@VS_2				
	1			
	12.884	0	0	
	-6.442	11.15787	0	
	0	0	17.8775	
	V	S	Hf	Li
	16	36	1	2
Direct				
	0.2057	0.212277	0.368941	
	0.485594	0.214781	0.368665	
	0.723849	0.227131	0.367692	
	0.945036	0.20816	0.365535	
	0.196828	0.434069	0.370962	
	0.435625	0.434822	0.369641	
	0.720362	0.458187	0.367758	
	0.969338	0.465388	0.365788	
	0.187	0.717762	0.359718	
	0.477817	0.747356	0.361273	
	0.742787	0.711071	0.358797	
	0.953911	0.695454	0.360843	
	0.231611	0.986896	0.36677	
	0.454962	0.965153	0.367536	
	0.704447	0.981971	0.365732	
	0.990085	0.973826	0.367661	
	0.039992	0.122959	0.44942	
	0.136053	0.051338	0.280576	
	0.303681	0.132119	0.449256	
	0.386242	0.055775	0.281996	
	0.551669	0.132702	0.451425	
	0.628665	0.054467	0.283669	
	0.793315	0.132438	0.452404	
	0.875697	0.043468	0.28941	
	0.038503	0.373999	0.451309	
	0.122759	0.289266	0.283783	
	0.281304	0.35721	0.45964	
	0.376148	0.291386	0.286737	
	0.548933	0.380319	0.448435	
	0.635746	0.300544	0.282185	
	0.800563	0.381872	0.451817	
	0.880863	0.298658	0.282972	
	0.042156	0.628765	0.448576	
	0.134661	0.54392	0.288789	
	0.287178	0.631165	0.433499	

	0.380179	0.548599	0.300243	
	0.547887	0.636088	0.44502	
	0.619585	0.540571	0.295042	
	0.803556	0.627453	0.44904	
	0.878995	0.539534	0.280108	
	0.051454	0.879246	0.44288	
	0.124597	0.800118	0.279086	
	0.289799	0.879413	0.445971	
	0.373778	0.800903	0.284763	
	0.545624	0.889887	0.451375	
	0.636897	0.801675	0.289849	
	0.799305	0.880737	0.435544	
	0.888783	0.797444	0.2784	
	0.36073	0.685787	0.621002	
	0.347921	0.532437	0.664773	
	0.651202	0.718098	0.648222	
	0.517969	0.548132	0.629448	
	0.369097	0.549934	0.51917	
	0.740116	0.731237	0.527933	
	0.572254	0.824133	0.572919	

Li_2S_6-Hf@VS_2				
	1			
	12.884	0	0	
	-6.442	11.15787	0	
	0	0	17.8775	
	V	S	Hf	Li
	16	38	1	2
Direct				
	0.20292	0.217139	0.362283	
	0.479824	0.219439	0.360621	
	0.716589	0.228962	0.359781	
	0.941551	0.214218	0.358705	
	0.192388	0.438917	0.365125	
	0.436522	0.43955	0.363224	
	0.711024	0.46082	0.358611	
	0.960815	0.467628	0.359381	
	0.182913	0.723856	0.354784	
	0.474176	0.751917	0.355908	
	0.739974	0.718476	0.353419	
	0.948868	0.699175	0.353448	
	0.228624	0.992478	0.359369	
	0.456159	0.975013	0.359488	
	0.700662	0.986171	0.359213	
	0.986264	0.978574	0.36051	
	0.036698	0.128725	0.4421	
	0.132488	0.056833	0.273362	
	0.299827	0.137507	0.442283	
	0.382141	0.059827	0.273925	
	0.547081	0.137011	0.444201	
	0.625577	0.059028	0.275389	
	0.78868	0.137609	0.444938	
	0.872505	0.049047	0.282495	
	0.033803	0.378661	0.444902	
	0.119384	0.295388	0.277624	
	0.279553	0.364346	0.452847	
	0.372465	0.29662	0.279183	
	0.545681	0.383362	0.442392	
	0.629701	0.303782	0.273767	
	0.794189	0.386603	0.443431	
	0.876424	0.303634	0.275834	
	0.036158	0.633949	0.442043	
	0.131027	0.549702	0.284828	
	0.281366	0.63789	0.433741	

	0.375671	0.553378	0.296114	
	0.552533	0.638512	0.436319	
	0.617036	0.547183	0.286059	
	0.798528	0.632046	0.441106	
	0.874286	0.543815	0.272608	
	0.04819	0.884257	0.435229	
	0.121217	0.804606	0.272627	
	0.288416	0.886475	0.43846	
	0.369801	0.803974	0.279962	
	0.542987	0.894881	0.444328	
	0.631307	0.806101	0.283062	
	0.797058	0.88738	0.428492	
	0.884623	0.802171	0.271046	
	0.630318	0.807976	0.627694	
	0.447322	0.722469	0.614887	
	0.591047	0.484829	0.700412	
	0.359166	0.541556	0.645666	
	0.844867	0.714537	0.640153	
	0.72604	0.538692	0.629057	
	0.376415	0.561705	0.509233	
	0.676704	0.698327	0.71252	
	0.727976	0.747638	0.532242	

Li_2S_8-Hf@VS_2				
	1			
	12.884	0	0	
	-6.442	11.15787	0	
	0	0	17.8775	
	V	S	Hf	Li
	16	40	1	2
Direct				
	0.200301	0.212975	0.362907	
	0.478715	0.217136	0.361213	
	0.714905	0.226603	0.361166	
	0.937666	0.210021	0.360008	
	0.188359	0.435971	0.364719	
	0.432556	0.437382	0.361941	
	0.71383	0.459068	0.36087	
	0.961643	0.464977	0.360958	
	0.181699	0.721002	0.354931	
	0.470667	0.75077	0.357004	
	0.737705	0.712677	0.354377	
	0.947137	0.696108	0.354549	
	0.226043	0.989783	0.359438	
	0.453098	0.972915	0.361228	
	0.698319	0.983326	0.360482	
	0.984524	0.975798	0.360888	
	0.034067	0.124567	0.442852	
	0.129578	0.053455	0.273456	
	0.297541	0.134912	0.442459	
	0.380594	0.058701	0.274669	
	0.544905	0.1363	0.445523	
	0.623097	0.056422	0.27721	
	0.786657	0.134588	0.446524	
	0.869441	0.046466	0.283049	
	0.03213	0.375138	0.446378	
	0.116381	0.291811	0.278668	
	0.275329	0.359518	0.452955	
	0.369348	0.293752	0.279727	
	0.543117	0.3829	0.441711	
	0.629891	0.3028	0.275215	
	0.793462	0.384041	0.445484	
	0.874639	0.300689	0.277232	
	0.034381	0.631229	0.442779	
	0.130536	0.547599	0.285487	
	0.280493	0.640283	0.439984	

	0.37472	0.553879	0.298283	
	0.556489	0.644219	0.444484	
	0.614542	0.543308	0.288317	
	0.797549	0.630998	0.443387	
	0.873838	0.540954	0.27383	
	0.046455	0.881797	0.435808	
	0.119107	0.801563	0.273568	
	0.284494	0.884279	0.439229	
	0.367897	0.803706	0.280464	
	0.537268	0.893353	0.446034	
	0.628641	0.802621	0.285798	
	0.796727	0.883147	0.427766	
	0.882118	0.798167	0.272171	
	0.843775	0.799054	0.651692	
	0.533118	0.502998	0.765222	
	0.868323	0.967655	0.64786	
	0.823859	0.741237	0.763642	
	0.404684	0.593441	0.642606	
	0.508853	0.507522	0.653677	
	0.702375	0.956216	0.637747	
	0.649898	0.682084	0.797475	
	0.373359	0.555035	0.504888	
	0.581413	0.777522	0.699652	
	0.664601	0.84938	0.52552	

S_8-Hf@VS_2			
	1		
	12.884	0	0
	-6.442	11.15787	0
	0	0	17.8775
	V	S	Hf
	16	40	1
Direct			
	0.214955	0.210868	0.366269
	0.479011	0.208117	0.363674
	0.717738	0.218937	0.363495
	0.947244	0.207323	0.364084
	0.208811	0.43392	0.366925
	0.444134	0.433617	0.365491
	0.699342	0.45047	0.360787
	0.972105	0.450521	0.362146
	0.191047	0.715351	0.35771
	0.479797	0.745407	0.354687
	0.744514	0.71242	0.353422
	0.953376	0.684417	0.354857
	0.228741	0.982827	0.363656
	0.472346	0.973111	0.360217
	0.714969	0.97557	0.35994
	0.9752	0.982375	0.363419
	0.042343	0.125421	0.447936
	0.133691	0.047787	0.279381
	0.30308	0.126409	0.447944
	0.384962	0.049676	0.277757
	0.55198	0.12869	0.447504
	0.633623	0.051538	0.277799
	0.794241	0.128339	0.447745
	0.881385	0.048436	0.278733
	0.043572	0.371648	0.448852
	0.128433	0.287705	0.282773
	0.287059	0.35507	0.455854
	0.377033	0.287368	0.2821
	0.548303	0.372956	0.447218
	0.630816	0.2938	0.277349
	0.799508	0.380711	0.442143
	0.881913	0.293965	0.278686
	0.042773	0.624943	0.443348
	0.138931	0.541471	0.287294
	0.294034	0.634386	0.439204

	0.381403	0.545676	0.29409
	0.561862	0.636137	0.435739
	0.620797	0.540215	0.284883
	0.801403	0.624038	0.441005
	0.876762	0.531086	0.27646
	0.047447	0.87616	0.429988
	0.126453	0.794624	0.276005
	0.295659	0.878267	0.438727
	0.37759	0.799402	0.280262
	0.550995	0.887162	0.443728
	0.638608	0.798817	0.281826
	0.799452	0.875834	0.434588
	0.89068	0.794269	0.274188
	0.379089	0.47258	0.68283
	0.758392	0.830443	0.670894
	0.480586	0.785791	0.68451
	0.354472	0.611289	0.642749
	0.488498	0.449342	0.605022
	0.66842	0.539968	0.642474
	0.763416	0.70747	0.598228
	0.633485	0.866982	0.623598
	0.389912	0.550247	0.50606

Li_2S-Rh@VS_2				
	1			
	12.884	0	0	
	-6.442	11.15787	0	
	0	0	17.8775	
	V	S	Rh	Li
	16	33	1	2
Direct				
	0.208785	0.191939	0.359167	
	0.461949	0.224462	0.362557	
	0.700283	0.22598	0.363013	
	0.960986	0.187423	0.348605	
	0.199449	0.484209	0.378397	
	0.489173	0.472696	0.358077	
	0.713286	0.464304	0.361202	
	0.942619	0.473819	0.369324	
	0.205658	0.730465	0.373685	
	0.484866	0.712458	0.350748	
	0.731268	0.715647	0.360665	
	0.962111	0.722205	0.362613	
	0.221487	0.967484	0.362389	
	0.461451	0.98565	0.36428	
	0.694709	0.975317	0.360354	
	0.967998	0.949439	0.360917	
	0.040835	0.122846	0.439943	
	0.130919	0.04323	0.275405	
	0.295893	0.133602	0.445818	
	0.379341	0.055052	0.27954	
	0.545203	0.139573	0.448573	
	0.623296	0.058105	0.277812	
	0.800043	0.136276	0.435583	
	0.875633	0.038413	0.2737	
	0.026285	0.374163	0.446797	
	0.132638	0.310717	0.290668	
	0.296692	0.38425	0.430784	
	0.377029	0.303509	0.281343	
	0.548844	0.387274	0.445661	
	0.626982	0.304147	0.278186	
	0.786039	0.383074	0.4506	
	0.881237	0.302829	0.292315	
	0.035122	0.635869	0.457492	
	0.124832	0.556068	0.292646	
	0.320988	0.663432	0.443078	

	0.374237	0.549022	0.285499	
	0.555842	0.637475	0.441063	
	0.636952	0.552174	0.274028	
	0.799116	0.634556	0.445358	
	0.879178	0.550783	0.279131	
	0.04778	0.881751	0.445982	
	0.133484	0.800308	0.28199	
	0.299021	0.893518	0.451547	
	0.377642	0.800871	0.294453	
	0.547257	0.887453	0.440429	
	0.640276	0.808391	0.2765	
	0.795997	0.88453	0.433583	
	0.884909	0.795511	0.273674	
	0.195678	0.465878	0.600086	
	0.148315	0.548482	0.504722	
	0.139293	0.289958	0.529575	
	0.376032	0.529745	0.531414	

Li_2S_2-Rh@VS_2				
	1			
	12.884	0	0	
	-6.442	11.15787	0	
	0	0	17.8775	
	V	S	Rh	Li
	16	34	1	2
Direct				
	0.209824	0.209334	0.363119	
	0.474183	0.213996	0.364717	
	0.710257	0.207657	0.360981	
	0.938387	0.191132	0.350939	
	0.223108	0.452921	0.367869	
	0.452786	0.443317	0.363054	
	0.697366	0.457863	0.362633	
	0.949024	0.473087	0.373752	
	0.199062	0.724664	0.371619	
	0.48914	0.743912	0.350813	
	0.73239	0.736172	0.361578	
	0.950057	0.711469	0.362752	
	0.231899	0.985003	0.364409	
	0.465828	0.972217	0.361793	
	0.705582	0.970697	0.356041	
	0.980794	0.964294	0.36147	
	0.038805	0.128549	0.440151	
	0.136683	0.049505	0.277656	
	0.300453	0.135507	0.447381	
	0.384209	0.052991	0.279103	
	0.549531	0.132381	0.446877	
	0.626543	0.052532	0.275106	
	0.792504	0.128333	0.442292	
	0.876178	0.042192	0.273503	
	0.03031	0.364775	0.445283	
	0.127273	0.296952	0.28459	
	0.296988	0.372737	0.450694	
	0.379701	0.294011	0.280366	
	0.544391	0.379616	0.448583	
	0.63059	0.299556	0.280443	
	0.787446	0.3799	0.448619	
	0.882176	0.308515	0.285244	
	0.031003	0.645827	0.454822	
	0.129009	0.547323	0.3034	
	0.312036	0.642609	0.445583	

	0.37982	0.547684	0.287317	
	0.547688	0.638677	0.432271	
	0.624709	0.548093	0.285457	
	0.787739	0.635329	0.443557	
	0.876154	0.547316	0.283813	
	0.05052	0.888321	0.444315	
	0.128827	0.800015	0.286049	
	0.299523	0.890015	0.448092	
	0.368969	0.796298	0.28603	
	0.550951	0.886554	0.441877	
	0.639456	0.806544	0.271373	
	0.801308	0.890791	0.438811	
	0.884474	0.80178	0.275961	
	0.253319	0.439395	0.634839	
	0.158022	0.521839	0.6135	
	0.126075	0.54905	0.496026	
	0.399137	0.557599	0.533934	
	0.125232	0.292185	0.538085	

Li_2S_4-Rh@VS_2				
	1			
	12.884	0	0	
	-6.442	11.15787	0	
	0	0	17.8775	
	V	S	Rh	Li
	16	36	1	2
Direct				
	0.20351	0.201745	0.365347	
	0.462759	0.207571	0.36692	
	0.693087	0.192527	0.364036	
	0.933568	0.188661	0.355996	
	0.189864	0.435838	0.377835	
	0.440083	0.439267	0.363675	
	0.695957	0.442263	0.366667	
	0.967008	0.471434	0.37634	
	0.199507	0.72235	0.372945	
	0.487871	0.739648	0.351387	
	0.729906	0.733152	0.36161	
	0.947058	0.699451	0.364178	
	0.224822	0.978329	0.366754	
	0.450192	0.958993	0.364581	
	0.703553	0.968129	0.356961	
	0.978326	0.959949	0.364473	
	0.034811	0.123056	0.442539	
	0.131769	0.043178	0.279545	
	0.293438	0.130866	0.449829	
	0.375963	0.043799	0.281434	
	0.539255	0.123733	0.449322	
	0.618305	0.043091	0.276523	
	0.784213	0.12024	0.445559	
	0.873221	0.040139	0.27669	
	0.022386	0.355376	0.455547	
	0.120043	0.291627	0.286652	
	0.288133	0.36813	0.452232	
	0.371793	0.287037	0.28244	
	0.538341	0.374683	0.448779	
	0.622538	0.28913	0.282077	
	0.786009	0.370863	0.44729	
	0.874051	0.300487	0.290657	
	0.024804	0.639225	0.457227	
	0.125788	0.542766	0.297968	
	0.302181	0.628597	0.436122	

	0.369866	0.541016	0.29389	
	0.543624	0.631884	0.432862	
	0.622108	0.541041	0.291851	
	0.782717	0.627484	0.443291	
	0.874285	0.538856	0.287485	
	0.045593	0.883175	0.446847	
	0.12417	0.793709	0.287471	
	0.293135	0.883206	0.452017	
	0.364475	0.789753	0.28604	
	0.546355	0.879631	0.441922	
	0.636582	0.801321	0.271091	
	0.797226	0.884235	0.441449	
	0.882341	0.796275	0.278134	
	0.449095	0.805581	0.632618	
	0.273042	0.733243	0.656003	
	0.301766	0.489484	0.630561	
	0.178641	0.541729	0.616989	
	0.124754	0.549532	0.494866	
	0.440688	0.653858	0.541464	
	0.383583	0.650425	0.719449	

Li_2S_6-Rh@VS_2				
	1			
	12.884	0	0	
	-6.442	11.15787	0	
	0	0	17.8775	
	V	S	Rh	Li
	16	38	1	2
Direct				
	0.215183	0.212111	0.358642	
	0.468593	0.223211	0.359941	
	0.697923	0.207341	0.358749	
	0.939782	0.196608	0.349069	
	0.198241	0.446608	0.367942	
	0.444307	0.45592	0.358078	
	0.692933	0.461391	0.357871	
	0.985219	0.480595	0.373838	
	0.199904	0.731251	0.362919	
	0.50221	0.734336	0.355095	
	0.737607	0.740085	0.357703	
	0.949664	0.706467	0.356032	
	0.233414	0.988935	0.358416	
	0.455686	0.965843	0.353699	
	0.707056	0.973707	0.351612	
	0.987825	0.970149	0.357357	
	0.042413	0.132697	0.434909	
	0.140211	0.055169	0.271984	
	0.300017	0.139895	0.44376	
	0.38553	0.057314	0.273483	
	0.54385	0.133234	0.441055	
	0.626936	0.052179	0.270678	
	0.789797	0.128547	0.43809	
	0.878079	0.048299	0.270355	
	0.034727	0.364257	0.450314	
	0.126199	0.299435	0.279339	
	0.293146	0.378823	0.446182	
	0.380053	0.301049	0.276463	
	0.542402	0.384528	0.442988	
	0.629492	0.301434	0.276083	
	0.794952	0.382798	0.434798	
	0.882814	0.312074	0.286161	
	0.039313	0.65242	0.449608	
	0.130538	0.552365	0.288051	
	0.308627	0.642675	0.419236	

	0.376221	0.549956	0.284248	
	0.555918	0.638506	0.43528	
	0.628173	0.552122	0.279887	
	0.791868	0.634578	0.439506	
	0.881575	0.546784	0.283443	
	0.054368	0.8928	0.439054	
	0.130587	0.805333	0.277505	
	0.298654	0.893005	0.441518	
	0.377862	0.79758	0.283079	
	0.550873	0.88708	0.436654	
	0.643945	0.806798	0.267851	
	0.806147	0.893873	0.434992	
	0.889279	0.805652	0.272358	
	0.201902	0.827827	0.619346	
	0.115676	0.663754	0.66838	
	0.33212	0.576594	0.659287	
	0.156872	0.543238	0.615269	
	0.519236	0.875885	0.651997	
	0.472503	0.711494	0.606029	
	0.139901	0.559818	0.487583	
	0.330674	0.814847	0.707061	
	0.392313	0.853258	0.549092	

Li_2S_8-Rh@VS_2				
	1			
	12.884	0	0	
	-6.442	11.15787	0	
	0	0	17.8775	
	V	S	Rh	Li
	16	40	1	2
Direct				
	0.218133	0.213349	0.361019	
	0.463537	0.215008	0.361483	
	0.698418	0.202146	0.35974	
	0.948903	0.197491	0.352056	
	0.208877	0.450844	0.37053	
	0.452895	0.457817	0.359464	
	0.692773	0.450256	0.359821	
	0.982756	0.475911	0.374671	
	0.207341	0.720324	0.368764	
	0.485019	0.735933	0.352512	
	0.720111	0.733601	0.357838	
	0.942734	0.707221	0.353692	
	0.22867	0.982752	0.362015	
	0.456129	0.965428	0.358729	
	0.706735	0.972434	0.354854	
	0.982917	0.969499	0.357975	
	0.042741	0.128899	0.43842	
	0.137014	0.051356	0.274984	
	0.298196	0.135541	0.446769	
	0.380039	0.050598	0.276244	
	0.544961	0.131434	0.443582	
	0.625713	0.050828	0.272739	
	0.792101	0.128708	0.440144	
	0.877831	0.046378	0.27321	
	0.035357	0.360739	0.450029	
	0.130632	0.300302	0.282184	
	0.296003	0.375732	0.448038	
	0.378182	0.296487	0.278225	
	0.542252	0.379967	0.44442	
	0.62507	0.294863	0.276586	
	0.794716	0.381211	0.437079	
	0.883851	0.30817	0.288012	
	0.035613	0.648396	0.448089	
	0.135541	0.550797	0.290621	
	0.318793	0.639849	0.445065	

	0.375114	0.547444	0.284482	
	0.549096	0.637278	0.435125	
	0.625791	0.546885	0.281491	
	0.788249	0.632385	0.437796	
	0.880468	0.543218	0.284121	
	0.047751	0.886323	0.439006	
	0.130822	0.799397	0.285501	
	0.299916	0.890438	0.445382	
	0.37383	0.797656	0.281126	
	0.549122	0.88629	0.439325	
	0.635839	0.805699	0.270196	
	0.79984	0.88913	0.436066	
	0.880593	0.803086	0.273414	
	0.492612	0.824643	0.653985	
	0.25432	0.464216	0.753417	
	0.451821	0.959525	0.659313	
	0.491956	0.757372	0.762107	
	0.137172	0.543191	0.618674	
	0.251071	0.475382	0.640554	
	0.268922	0.886243	0.64848	
	0.318713	0.640731	0.794386	
	0.130645	0.553784	0.48876	
	0.207502	0.704256	0.709612	
	0.310688	0.77724	0.554379	

S_8-Rh@VS_2				
	1			
	12.884	0	0	
	-6.442	11.15787	0	
	0	0	17.8775	
	V	S	Rh	
	16	40	1	
Direct				
	0.211754	0.209388	0.370968	
	0.464702	0.220427	0.373102	
	0.693738	0.205328	0.371874	
	0.937308	0.193834	0.362639	
	0.194041	0.444155	0.380761	
	0.440662	0.453211	0.370714	
	0.689192	0.459132	0.370107	
	0.981392	0.475694	0.385167	
	0.196583	0.727879	0.376468	
	0.498629	0.732557	0.370291	
	0.733372	0.737227	0.370443	
	0.944669	0.703092	0.365273	
	0.229466	0.984316	0.371362	
	0.450546	0.961251	0.369617	
	0.703529	0.971204	0.364864	
	0.985216	0.96768	0.369792	
	0.039296	0.129426	0.448014	
	0.137987	0.051886	0.284752	
	0.295852	0.13573	0.456405	
	0.382952	0.053225	0.287501	
	0.541064	0.131581	0.454753	
	0.622791	0.048611	0.284835	
	0.786591	0.126042	0.451198	
	0.87483	0.045312	0.284124	
	0.030584	0.360537	0.463	
	0.12314	0.29679	0.291917	
	0.289272	0.375353	0.457803	
	0.376901	0.298105	0.289293	
	0.53918	0.38167	0.455791	
	0.625263	0.297868	0.288879	
	0.792069	0.380427	0.44719	
	0.879137	0.307685	0.298402	
	0.032638	0.648273	0.460773	
	0.128605	0.55029	0.300408	
	0.304176	0.638499	0.432816	

	0.373574	0.548425	0.297525	
	0.553348	0.63549	0.448686	
	0.624058	0.548831	0.292083	
	0.789165	0.631592	0.450949	
	0.876868	0.542446	0.294396	
	0.050324	0.888207	0.451069	
	0.128575	0.802128	0.289907	
	0.294459	0.887747	0.457098	
	0.375448	0.795818	0.297178	
	0.549124	0.885812	0.45139	
	0.638994	0.80377	0.28163	
	0.802831	0.890189	0.448105	
	0.885006	0.80396	0.284186	
	0.329544	0.384012	0.682221	
	0.266648	0.719222	0.680579	
	0.083988	0.411922	0.687644	
	0.163651	0.331005	0.634574	
	0.459848	0.522794	0.620125	
	0.50735	0.683763	0.671208	
	0.417403	0.761562	0.621529	
	0.120816	0.569689	0.628329	
	0.132478	0.555233	0.499042	

Li_2S-Ta@VS_2				
	1			
	12.884	0	0	
	-6.442	11.15787	0	
	0	0	17.8775	
	V	S	Ta	Li
	16	33	1	2
Direct				
	0.216513	0.209753	0.364968	
	0.476496	0.216865	0.364617	
	0.716545	0.241919	0.366265	
	0.940979	0.220733	0.365218	
	0.211339	0.438428	0.356216	
	0.449266	0.439557	0.359157	
	0.711967	0.482455	0.365503	
	0.953932	0.46815	0.364899	
	0.191391	0.729526	0.364701	
	0.480535	0.761722	0.355807	
	0.744645	0.732736	0.359408	
	0.959779	0.707004	0.363424	
	0.193721	0.972204	0.368015	
	0.490829	0.997032	0.366222	
	0.722409	0.969238	0.366622	
	0.967439	0.99315	0.364959	
	0.046184	0.144296	0.445256	
	0.126615	0.051742	0.279323	
	0.295959	0.132584	0.447719	
	0.383329	0.05452	0.288009	
	0.555483	0.148736	0.452992	
	0.640664	0.066741	0.285568	
	0.797153	0.144791	0.448742	
	0.880256	0.061301	0.280192	
	0.0497	0.390081	0.441999	
	0.1256	0.292121	0.278653	
	0.294531	0.375365	0.447205	
	0.378138	0.291212	0.2787	
	0.545762	0.387581	0.44794	
	0.631267	0.311406	0.282142	
	0.798905	0.396596	0.450398	
	0.876335	0.310468	0.28012	
	0.042779	0.638294	0.449599	
	0.136552	0.555184	0.29288	
	0.295596	0.638343	0.433895	

	0.387607	0.562274	0.296963	
	0.555146	0.64146	0.450233	
	0.626666	0.560179	0.286997	
	0.80088	0.641669	0.448854	
	0.87667	0.553868	0.280584	
	0.040815	0.888466	0.444887	
	0.123817	0.806003	0.279488	
	0.297151	0.890998	0.439621	
	0.383552	0.820427	0.283958	
	0.560026	0.903586	0.452316	
	0.640465	0.810313	0.282301	
	0.804776	0.890626	0.446887	
	0.889713	0.809673	0.280127	
	0.383082	0.592207	0.618277	
	0.452901	0.711308	0.51499	
	0.210905	0.473065	0.532079	
	0.461357	0.477155	0.538684	

Li_2S_2-Ta@VS_2				
	1			
	12.884	0	0	
	-6.442	11.15787	0	
	0	0	17.8775	
	V	S	Ta	Li
	16	34	1	2
Direct				
	0.188315	0.199648	0.361917	
	0.474539	0.222553	0.359329	
	0.713796	0.215819	0.361917	
	0.956449	0.229667	0.359311	
	0.198995	0.436025	0.360975	
	0.455217	0.436712	0.356492	
	0.702798	0.456794	0.360321	
	0.954107	0.472429	0.361068	
	0.19418	0.732454	0.360255	
	0.466049	0.683053	0.346626	
	0.727625	0.726521	0.362291	
	0.942674	0.698887	0.362098	
	0.21517	0.984166	0.362364	
	0.444804	0.980625	0.363804	
	0.744445	0.988081	0.360885	
	0.967628	0.952547	0.361757	
	0.037113	0.126596	0.441211	
	0.123646	0.045736	0.27502	
	0.292611	0.137268	0.442153	
	0.37644	0.052227	0.276147	
	0.544655	0.135036	0.445983	
	0.624657	0.043248	0.289871	
	0.800921	0.13947	0.446037	
	0.885162	0.055016	0.277683	
	0.042952	0.383507	0.443313	
	0.118956	0.290861	0.274261	
	0.290252	0.372179	0.441543	
	0.374173	0.286726	0.276158	
	0.542828	0.375945	0.445446	
	0.629892	0.296663	0.276486	
	0.793297	0.382325	0.443681	
	0.875612	0.303639	0.277209	
	0.040806	0.633505	0.445173	
	0.13124	0.553055	0.290283	
	0.295496	0.623181	0.430858	

	0.370809	0.528608	0.272497	
	0.541202	0.618436	0.436729	
	0.625965	0.546509	0.283273	
	0.789826	0.628255	0.445416	
	0.871561	0.546147	0.276875	
	0.045119	0.879722	0.442626	
	0.120972	0.801892	0.278659	
	0.287557	0.888554	0.445226	
	0.378965	0.796271	0.298305	
	0.553126	0.893201	0.445296	
	0.63542	0.807507	0.28369	
	0.799976	0.885147	0.444045	
	0.877503	0.794851	0.27632	
	0.5682	0.699141	0.611935	
	0.342639	0.761913	0.60357	
	0.456306	0.726134	0.517727	
	0.371729	0.611867	0.662143	
	0.545047	0.862852	0.651382	

Li_2S_4-Ta@VS_2				
	1			
	12.884	0	0	
	-6.442	11.15787	0	
	0	0	17.8775	
	V	S	Ta	Li
	16	36	1	2
Direct				
	0.187216	0.202606	0.364072	
	0.475713	0.213315	0.364507	
	0.730014	0.23359	0.36416	
	0.954014	0.22446	0.362196	
	0.193862	0.436195	0.363315	
	0.43591	0.430871	0.360476	
	0.700245	0.458663	0.363039	
	0.966833	0.476746	0.36342	
	0.187112	0.726223	0.35998	
	0.485912	0.750969	0.352898	
	0.735917	0.730032	0.364713	
	0.944008	0.699542	0.361787	
	0.216073	0.984529	0.364815	
	0.433115	0.957804	0.362377	
	0.735199	0.990274	0.361277	
	0.970634	0.959913	0.365243	
	0.035697	0.127566	0.445423	
	0.12489	0.047493	0.277629	
	0.292871	0.136465	0.444313	
	0.375231	0.050638	0.278623	
	0.545264	0.13114	0.444452	
	0.624921	0.051681	0.293451	
	0.798887	0.141442	0.44842	
	0.883908	0.056964	0.280297	
	0.043016	0.384841	0.447072	
	0.120647	0.293776	0.276795	
	0.283859	0.369872	0.44682	
	0.369499	0.287514	0.279929	
	0.538565	0.378186	0.445694	
	0.629794	0.298367	0.279999	
	0.795483	0.38827	0.446658	
	0.881242	0.307584	0.27856	
	0.03842	0.635926	0.447916	
	0.132586	0.553765	0.287603	
	0.294058	0.640331	0.43536	

	0.377782	0.552153	0.297279	
	0.54693	0.635585	0.433059	
	0.620447	0.549986	0.290465	
	0.791096	0.630676	0.447119	
	0.872323	0.546226	0.279987	
	0.044664	0.881959	0.444831	
	0.117955	0.801523	0.278947	
	0.284271	0.884054	0.447757	
	0.367537	0.798278	0.280685	
	0.546467	0.892417	0.441096	
	0.641709	0.812105	0.277926	
	0.797323	0.887376	0.444594	
	0.881189	0.799364	0.277718	
	0.555932	0.862124	0.713533	
	0.56179	0.881628	0.596937	
	0.302161	0.586112	0.620268	
	0.444528	0.551309	0.606027	
	0.45259	0.701247	0.51988	
	0.47505	0.65926	0.716644	
	0.372173	0.801887	0.655659	

Li_2S_6-Ta@VS_2				
	1			
	12.884	0	0	
	-6.442	11.15787	0	
	0	0	17.8775	
	V	S	Ta	Li
	16	38	1	2
Direct				
	0.210539	0.202895	0.363869	
	0.47066	0.23341	0.36253	
	0.698062	0.215736	0.362631	
	0.933816	0.214395	0.360651	
	0.179136	0.430747	0.35587	
	0.424746	0.445804	0.36025	
	0.721957	0.462473	0.36261	
	0.963966	0.465546	0.362722	
	0.186634	0.729199	0.358728	
	0.485525	0.712221	0.349675	
	0.724779	0.702626	0.358302	
	0.944017	0.700463	0.359453	
	0.187828	0.960756	0.362207	
	0.425779	0.975337	0.360246	
	0.723378	0.983777	0.36116	
	0.96924	0.978599	0.364307	
	0.037104	0.133659	0.4428	
	0.118473	0.048309	0.276272	
	0.287814	0.128939	0.443702	
	0.362396	0.052683	0.27735	
	0.531387	0.130474	0.443955	
	0.610426	0.041811	0.287932	
	0.78429	0.132368	0.447679	
	0.873058	0.053025	0.277361	
	0.033601	0.375126	0.445703	
	0.109041	0.28492	0.278209	
	0.276234	0.372533	0.44514	
	0.368145	0.293277	0.277197	
	0.538774	0.382551	0.442698	
	0.62411	0.300995	0.277774	
	0.789743	0.378098	0.446513	
	0.866382	0.29898	0.279064	
	0.036983	0.631911	0.444551	
	0.128272	0.551645	0.286404	
	0.277136	0.627206	0.478806	

	0.359404	0.542497	0.281647	
	0.540056	0.617529	0.439387	
	0.617272	0.539784	0.287908	
	0.79293	0.625324	0.446807	
	0.874857	0.543919	0.277174	
	0.031045	0.879279	0.442996	
	0.113936	0.799804	0.275044	
	0.275608	0.881638	0.445894	
	0.369578	0.793569	0.296598	
	0.532297	0.880775	0.428739	
	0.639352	0.800861	0.277257	
	0.784071	0.875384	0.436636	
	0.87653	0.796987	0.278154	
	0.698574	-0.02737	0.635338	
	0.520401	0.847967	0.629936	
	0.757827	0.675309	0.671227	
	0.487041	0.672158	0.640153	
	0.972063	0.954427	0.636296	
	0.900167	0.779281	0.609079	
	0.466855	0.719579	0.513289	
	0.779735	0.867433	0.69952	
	0.862092	0.008251	0.539879	

Li_2S_8-Ta@VS_2				
	1			
	12.884	0	0	
	-6.442	11.15787	0	
	0	0	17.8775	
	V	S	Ta	Li
	16	40	1	2
Direct				
	0.215314	0.203728	0.358868	
	0.481386	0.233607	0.354673	
	0.711194	0.220679	0.356805	
	0.948137	0.222424	0.355741	
	0.186466	0.435367	0.355981	
	0.433652	0.44292	0.356276	
	0.729947	0.469426	0.356457	
	0.964605	0.463083	0.357116	
	0.19452	0.731761	0.356775	
	0.466698	0.707911	0.366087	
	0.739516	0.724785	0.352891	
	0.956346	0.701959	0.354252	
	0.190163	0.960036	0.357273	
	0.444876	0.985179	0.35288	
	0.741659	0.986255	0.358571	
	0.974187	0.986756	0.358907	
	0.046873	0.137754	0.438956	
	0.125866	0.050803	0.27069	
	0.296255	0.13023	0.436488	
	0.373245	0.057152	0.272193	
	0.543251	0.137725	0.438337	
	0.623848	0.044899	0.284558	
	0.795382	0.137994	0.442383	
	0.882877	0.057069	0.27163	
	0.036336	0.376917	0.44288	
	0.120247	0.292478	0.274028	
	0.284567	0.372129	0.441601	
	0.3756	0.296176	0.272182	
	0.547292	0.385905	0.435936	
	0.635397	0.306654	0.27154	
	0.796343	0.38376	0.440706	
	0.875792	0.302024	0.272106	
	0.042385	0.6338	0.440537	
	0.134866	0.553631	0.283292	
	0.279777	0.626747	0.452676	

	0.369244	0.54805	0.2843	
	0.547706	0.618839	0.448517	
	0.623869	0.550746	0.288465	
	0.802907	0.635331	0.439426	
	0.882679	0.547913	0.270562	
	0.038326	0.882923	0.437781	
	0.123133	0.801672	0.269815	
	0.288391	0.889249	0.438909	
	0.379067	0.799419	0.292356	
	0.550457	0.899007	0.445166	
	0.62989	0.801606	0.288591	
	0.801239	0.886407	0.438211	
	0.885591	0.803563	0.272932	
	0.42447	0.341886	0.60454	
	0.468661	0.681764	0.714184	
	0.538622	0.281717	0.635275	
	0.34682	0.372253	0.699242	
	0.723294	0.715528	0.627013	
	0.590698	0.755051	0.620426	
	0.711038	0.424748	0.636067	
	0.46873	0.532192	0.750975	
	0.464522	0.722463	0.506815	
	0.665506	0.548919	0.708336	
	0.661846	0.533529	0.54237	

S_8-Ta@VS_2			
	1		
	12.884	0	0
	-6.442	11.15787	0
	0	0	17.8775
	V	S	Ta
	16	40	1
Direct			
	0.197114	0.200421	0.360977
	0.481083	0.220468	0.358078
	0.718093	0.218037	0.358593
	0.954395	0.221733	0.356805
	0.186185	0.429002	0.359169
	0.443594	0.432364	0.353788
	0.712864	0.457919	0.35951
	0.955396	0.457933	0.35939
	0.190325	0.722268	0.360571
	0.435817	0.730983	0.355259
	0.742894	0.737028	0.352962
	0.952509	0.693041	0.358055
	0.207473	0.975919	0.362421
	0.450242	0.981994	0.359914
	0.748147	0.990748	0.357722
	0.977307	0.975304	0.360959
	0.043307	0.131508	0.442986
	0.128862	0.048399	0.273044
	0.295859	0.134649	0.440072
	0.377025	0.056018	0.275097
	0.547641	0.137546	0.443954
	0.628212	0.045039	0.284969
	0.799266	0.137318	0.444437
	0.888504	0.055319	0.272248
	0.037213	0.376052	0.444619
	0.121358	0.289101	0.272665
	0.290427	0.373924	0.440916
	0.373625	0.287396	0.274713
	0.542636	0.38088	0.440796
	0.632372	0.29859	0.273905
	0.79257	0.380923	0.443225
	0.876591	0.299613	0.273328
	0.0347	0.627698	0.444817
	0.129158	0.546999	0.286185
	0.2772	0.62236	0.435223

	0.376684	0.548859	0.293082
	0.545252	0.630101	0.470932
	0.623287	0.55237	0.289847
	0.791126	0.632441	0.439129
	0.874687	0.541887	0.274165
	0.043345	0.880096	0.439401
	0.118462	0.797712	0.276471
	0.290846	0.884755	0.442721
	0.362608	0.809072	0.277543
	0.552715	0.895389	0.429398
	0.624033	0.797842	0.293647
	0.803144	0.886675	0.440671
	0.888578	0.802827	0.273985
	0.327026	0.614893	0.623526
	0.360983	0.483076	0.681121
	0.408829	0.784371	0.674107
	0.495109	0.469208	0.634678
	0.572795	0.874359	0.615377
	0.654454	0.580937	0.690248
	0.719573	0.864469	0.667713
	0.74237	0.73332	0.624016
	0.455189	0.725481	0.509009

Li_2S-Y@VS_2				
	1			
	12.884	0	0	
	-6.442	11.15787	0	
	0	0	17.8775	
	V	S	Y	Li
	16	33	1	2
Direct				
	0.187718	0.196808	0.357623	
	0.474928	0.210453	0.358654	
	0.715933	0.214616	0.357816	
	0.947666	0.215991	0.356348	
	0.189009	0.432375	0.358206	
	0.434647	0.432359	0.35322	
	0.706889	0.452555	0.358819	
	0.959109	0.463543	0.358887	
	0.186322	0.720858	0.359186	
	0.484923	0.747046	0.355205	
	0.733679	0.722696	0.362702	
	0.943435	0.691543	0.357729	
	0.217166	0.978839	0.360128	
	0.44246	0.964766	0.360021	
	0.731443	0.982143	0.356675	
	0.972052	0.95477	0.36106	
	0.035371	0.121805	0.439162	
	0.124094	0.039956	0.272742	
	0.292904	0.132455	0.43872	
	0.376199	0.047995	0.274055	
	0.544164	0.12956	0.442316	
	0.624142	0.044413	0.283057	
	0.795661	0.13383	0.443418	
	0.881217	0.04906	0.275469	
	0.035398	0.374062	0.442952	
	0.117124	0.288191	0.271826	
	0.28113	0.362172	0.440211	
	0.369245	0.285256	0.274278	
	0.539582	0.374695	0.4391	
	0.628239	0.293989	0.274164	
	0.791025	0.377224	0.44149	
	0.874413	0.297604	0.273378	
	0.034682	0.625231	0.443471	
	0.128662	0.547108	0.284288	
	0.288308	0.628503	0.431356	

	0.376423	0.550062	0.291833	
	0.544465	0.629657	0.433662	
	0.620486	0.544651	0.288036	
	0.791512	0.623693	0.443488	
	0.87171	0.539413	0.274476	
	0.045722	0.875622	0.4396	
	0.117983	0.794746	0.275665	
	0.286562	0.879872	0.443192	
	0.369287	0.796145	0.28251	
	0.552824	0.897203	0.440048	
	0.636844	0.803094	0.276896	
	0.798394	0.882392	0.440958	
	0.878107	0.791735	0.275059	
	0.365969	0.52717	0.615325	
	0.451104	0.705914	0.534214	
	0.203826	0.452638	0.531055	
	0.451994	0.451059	0.53072	

Li_2S_2-Y@VS_2				
	1			
	12.884	0	0	
	-6.442	11.15787	0	
	0	0	17.8775	
	V	S	Y	Li
	16	34	1	2
Direct				
	0.190674	0.187443	0.364494	
	0.447792	0.196976	0.36269	
	0.700787	0.210206	0.361021	
	0.941305	0.197775	0.361809	
	0.180453	0.433586	0.364993	
	0.438087	0.428941	0.366299	
	0.696184	0.448613	0.36482	
	0.941642	0.44776	0.364362	
	0.179606	0.700644	0.365693	
	0.46207	0.735181	0.348046	
	0.719613	0.70463	0.36322	
	0.947531	0.699898	0.365816	
	0.193449	0.949792	0.36551	
	0.456241	0.970354	0.356944	
	0.706067	0.969255	0.3568	
	0.953583	0.953575	0.365124	
	0.02895	0.118217	0.445108	
	0.115428	0.03627	0.279424	
	0.282461	0.118023	0.445155	
	0.363335	0.042039	0.27822	
	0.534851	0.124612	0.444318	
	0.620357	0.048575	0.276493	
	0.783694	0.125079	0.443529	
	0.871785	0.041684	0.278282	
	0.028246	0.368142	0.447436	
	0.115897	0.286055	0.281397	
	0.271841	0.354204	0.447401	
	0.362191	0.285008	0.281013	
	0.532994	0.368744	0.448077	
	0.615679	0.292017	0.281247	
	0.783036	0.372138	0.447412	
	0.867476	0.29184	0.280656	
	0.029424	0.620184	0.448787	
	0.117793	0.538362	0.285847	
	0.28587	0.626702	0.437655	

	0.367798	0.540318	0.293681	
	0.536032	0.628339	0.431492	
	0.614078	0.53764	0.286911	
	0.785317	0.621886	0.448242	
	0.866702	0.538712	0.284166	
	0.033502	0.87284	0.446751	
	0.11235	0.787795	0.281426	
	0.28568	0.875694	0.441568	
	0.361414	0.795267	0.277425	
	0.53963	0.881806	0.43699	
	0.625502	0.794172	0.278002	
	0.783758	0.875831	0.44077	
	0.872149	0.788704	0.280371	
	0.53907	0.614065	0.641444	
	0.47398	0.733637	0.62419	
	0.380048	0.540465	0.535716	
	0.653258	0.755342	0.544616	
	0.379872	0.798465	0.524949	

Li_2S_4-Y@VS_2				
	1			
	12.884	0	0	
	-6.442	11.15787	0	
	0	0	17.8775	
	V	S	Y	Li
	16	36	1	2
Direct				
	0.182986	0.183753	0.357927	
	0.484117	0.224254	0.355296	
	0.719759	0.221667	0.358449	
	0.949921	0.215847	0.358551	
	0.181242	0.463444	0.352543	
	0.481645	0.463033	0.348566	
	0.725128	0.469061	0.356708	
	0.947349	0.459763	0.357824	
	0.187642	0.725395	0.361364	
	0.479407	0.750885	0.351592	
	0.735793	0.738028	0.360434	
	0.95281	0.714035	0.357193	
	0.187022	0.952882	0.358237	
	0.479065	0.989343	0.356519	
	0.723325	0.982884	0.358717	
	0.953927	0.966006	0.358253	
	0.036456	0.129241	0.443374	
	0.117038	0.041723	0.270797	
	0.293814	0.12936	0.431885	
	0.378254	0.05434	0.280357	
	0.551203	0.141456	0.442832	
	0.635003	0.058087	0.274955	
	0.795402	0.138332	0.444534	
	0.879672	0.056521	0.27476	
	0.039199	0.385749	0.441922	
	0.126777	0.299	0.279604	
	0.288037	0.373928	0.42769	
	0.372997	0.295481	0.281889	
	0.551304	0.388277	0.437317	
	0.636176	0.304307	0.272974	
	0.794296	0.385148	0.443967	
	0.878399	0.302146	0.273579	
	0.034423	0.633592	0.441483	
	0.120906	0.555806	0.274548	
	0.288144	0.64436	0.431352	

	0.378663	0.556023	0.293296	
	0.553897	0.641986	0.431818	
	0.63864	0.558433	0.274729	
	0.7939	0.6342	0.440094	
	0.879804	0.551903	0.272327	
	0.036054	0.88091	0.44099	
	0.118745	0.800341	0.271847	
	0.291151	0.889585	0.435386	
	0.375105	0.809154	0.280996	
	0.548802	0.894217	0.440623	
	0.637184	0.810009	0.276298	
	0.795754	0.890073	0.443299	
	0.880074	0.805141	0.272405	
	0.398658	0.245334	0.612707	
	0.496336	0.391566	0.678853	
	0.267424	0.446722	0.649322	
	0.44954	0.530181	0.660469	
	0.367963	0.544268	0.520524	
	0.587496	0.359485	0.562156	
	0.241171	0.228024	0.530649	

Li_2S_6-Y@VS_2				
	1			
	12.884	0	0	
	-6.442	11.15787	0	
	0	0	17.8775	
	V	S	Y	Li
	16	38	1	2
Direct				
	0.18592	0.179394	0.364975	
	0.476869	0.211603	0.355167	
	0.704364	0.189384	0.360911	
	0.951674	0.211423	0.361508	
	0.192454	0.47553	0.360742	
	0.456507	0.432312	0.352881	
	0.711006	0.470237	0.359847	
	0.932619	0.437049	0.36185	
	0.207325	0.720762	0.362763	
	0.439052	0.72308	0.36067	
	0.733349	0.730914	0.354866	
	0.957204	0.690313	0.362153	
	0.182628	0.936214	0.363345	
	0.460571	0.970436	0.356008	
	0.708434	0.952871	0.357938	
	0.951985	0.969155	0.359159	
	0.031424	0.119285	0.446393	
	0.115322	0.035517	0.27712	
	0.286825	0.118049	0.438633	
	0.368467	0.041054	0.280374	
	0.541235	0.1211	0.442134	
	0.620509	0.03545	0.275631	
	0.78665	0.118143	0.444095	
	0.870598	0.043	0.276487	
	0.031921	0.372013	0.444147	
	0.123644	0.293106	0.288702	
	0.280913	0.366017	0.434648	
	0.36754	0.278102	0.276253	
	0.538151	0.371077	0.441157	
	0.628937	0.290235	0.27756	
	0.77983	0.366928	0.441694	
	0.867308	0.283984	0.276892	
	0.033857	0.617446	0.443101	
	0.112923	0.542686	0.28012	
	0.280609	0.632857	0.448688	

	0.364449	0.536375	0.291659	
	0.542684	0.62954	0.419656	
	0.622981	0.544123	0.279376	
	0.789997	0.62887	0.442704	
	0.868598	0.53547	0.276687	
	0.029286	0.867575	0.441096	
	0.115304	0.786434	0.276326	
	0.285883	0.8792	0.442742	
	0.365192	0.795172	0.274719	
	0.537244	0.876813	0.441238	
	0.615984	0.786886	0.281327	
	0.789819	0.876881	0.4436	
	0.879427	0.796326	0.274852	
	0.66023	0.292451	0.611952	
	0.50573	0.163474	0.659816	
	0.339376	0.291838	0.652237	
	0.420375	0.229172	0.726668	
	0.578418	0.576112	0.609423	
	0.422453	0.477064	0.670162	
	0.395275	0.540948	0.524054	
	0.468448	0.206269	0.534064	
	0.675351	0.466311	0.547091	

Li_2S_8-Y@VS_2				
	1			
	12.884	0	0	
	-6.442	11.15787	0	
	0	0	17.8775	
	V	S	Y	Li
	16	40	1	2
Direct				
	0.178683	0.183604	0.356705	
	0.44834	0.203698	0.355533	
	0.696094	0.207172	0.354612	
	0.935393	0.202227	0.354763	
	0.166232	0.450319	0.347777	
	0.463562	0.450043	0.348829	
	0.695176	0.442952	0.351354	
	0.928012	0.446547	0.35124	
	0.187281	0.734748	0.357299	
	0.453348	0.74269	0.348661	
	0.730646	0.739246	0.356122	
	0.96141	0.736995	0.354212	
	0.205242	0.970479	0.352386	
	0.458751	0.979822	0.353916	
	0.699377	0.975347	0.354236	
	0.946471	0.969393	0.352938	
	0.025011	0.127633	0.438653	
	0.111434	0.04384	0.268148	
	0.281413	0.128576	0.437431	
	0.367236	0.0491	0.269286	
	0.532534	0.131135	0.440185	
	0.614728	0.048192	0.269374	
	0.778949	0.129981	0.440031	
	0.86005	0.046831	0.270167	
	0.022911	0.377671	0.436489	
	0.110049	0.289757	0.27492	
	0.272835	0.368388	0.427421	
	0.359201	0.290369	0.276084	
	0.533536	0.376623	0.438428	
	0.611949	0.28973	0.270153	
	0.778633	0.374189	0.438801	
	0.859254	0.290693	0.270451	
	0.025643	0.633861	0.431842	
	0.108111	0.550881	0.274834	
	0.27094	0.638901	0.429095	

	0.363202	0.547491	0.291027	
	0.544871	0.638769	0.427495	
	0.622008	0.550024	0.275918	
	0.787929	0.634306	0.430249	
	0.862081	0.546311	0.275057	
	0.035138	0.888883	0.435389	
	0.115335	0.805785	0.267509	
	0.283144	0.890191	0.436932	
	0.361426	0.804138	0.271595	
	0.531336	0.886821	0.437885	
	0.61751	0.798306	0.276495	
	0.786846	0.889492	0.438225	
	0.871706	0.806155	0.267297	
	0.626765	0.428057	0.770285	
	0.440297	0.634485	0.796035	
	0.792087	0.485702	0.723763	
	0.635294	0.540681	0.858775	
	0.473098	0.533344	0.63335	
	0.366437	0.487494	0.728628	
	0.819019	0.598548	0.633801	
	0.620635	0.679095	0.81179	
	0.366262	0.54817	0.510887	
	0.669642	0.628141	0.6845	
	0.713863	0.479888	0.528396	

S_8-Y@VS_2			
	1		
	12.884	0	0
	-6.442	11.15787	0
	0	0	17.8775
	V	S	Y
	16	40	1
Direct			
	0.197833	0.178408	0.359643
	0.468086	0.208605	0.356976
	0.704417	0.214773	0.357461
	0.940332	0.21166	0.355076
	0.17743	0.450738	0.350518
	0.473951	0.447079	0.349488
	0.710432	0.45069	0.356036
	0.942653	0.458441	0.358158
	0.197142	0.729484	0.362229
	0.471273	0.743472	0.35104
	0.74485	0.725052	0.35881
	0.962033	0.709284	0.3566
	0.216975	0.963538	0.357295
	0.462818	0.977402	0.358559
	0.708046	0.980515	0.355177
	0.970353	0.953015	0.357274
	0.037915	0.122728	0.434976
	0.126897	0.037763	0.270509
	0.295121	0.124204	0.441341
	0.378725	0.048588	0.27284
	0.544	0.131846	0.444076
	0.623609	0.050882	0.273194
	0.789153	0.13149	0.441688
	0.874312	0.046434	0.277859
	0.034009	0.376036	0.439657
	0.121212	0.288348	0.279856
	0.283591	0.364408	0.42703
	0.372132	0.287911	0.276049
	0.544989	0.376506	0.440157
	0.625218	0.294102	0.271603
	0.788647	0.377028	0.443407
	0.870023	0.297008	0.273831
	0.038123	0.627391	0.441239
	0.119716	0.551148	0.277538
	0.286487	0.63465	0.433629

	0.374227	0.546934	0.293478
	0.559677	0.638305	0.426741
	0.633163	0.549059	0.275835
	0.798682	0.62734	0.439606
	0.87286	0.543958	0.273273
	0.046511	0.876638	0.439181
	0.126244	0.799021	0.272738
	0.295677	0.885212	0.442972
	0.371057	0.801704	0.277541
	0.545223	0.886511	0.440588
	0.632622	0.800378	0.279515
	0.799555	0.884298	0.434376
	0.886004	0.797282	0.26987
	0.170764	0.408712	0.619157
	0.225784	0.292731	0.659109
	0.225981	0.556176	0.689452
	0.38423	0.336196	0.602763
	0.38217	0.694354	0.643058
	0.532793	0.446159	0.668421
	0.534041	0.710062	0.694793
	0.592243	0.61742	0.627675
	0.383555	0.542094	0.511367
